# Differential structural cortical correlates of positive, negative, and linguistic control formal thought disorder dimensions in schizophrenia

**DOI:** 10.1038/s41537-025-00644-8

**Published:** 2025-07-16

**Authors:** Jürgen Hänggi, Sebastian Walther, Nicole Gangl, Frauke Conring, Katharina Stegmayer

**Affiliations:** 1 Translational Research Center, University Hospital of Psychiatry and Psychotherapy, University of Bern, Bern, Switzerland; 2 Translational Imaging Center, Swiss Institute for Translational and Entrepreneurial Medicine, Bern, Switzerland; 3 Department of Psychiatry, Psychosomatics, and Psychotherapy, Center for Mental Health, University of Würzburg, Würzburg, Germany

**Keywords:** Schizophrenia, Psychosis, Biomarkers, Psychosis

## Abstract

Formal thought disorder (FTD) is a core symptom of schizophrenia. The pathophysiology of FTD is still unclear. We focus on multiple cortical measures to capture the exact nature of brain alterations (e.g., plasticity, early brain development) in FTD dimensions. We included 70 schizophrenia patients. We assessed FTD, acquired structural neuroimaging scans, and analyzed cortical thickness, volume, surface area, and local gyrification (IGI). Results reveal negative FTD to be associated with different structural brain correlates compared to the positive and linguistic control FTD dimensions most prominent in markers of early brain development. Severity of positive and linguistic control FTD dimensions correlated positively with IGI of core language regions including temporal, Heschl’s, and inferior frontal gyri. Severity of negative FTD dimension was inversely correlated with lGI of occipital and parietal regions. Findings propose distinguishable changes most prominent in markers of early brain development associated with FTD dimensions suggesting a distinct pathophysiology.

## Introduction

Formal thought disorder (FTD) describes a multidimensional syndrome evident in multiple psychiatric conditions^1,2^. FTD is a core symptom in schizophrenia spectrum disorders with up to 91% prevalence^3^. FTD occurs early in the course of schizophrenia^4–6^. In general, FTD significantly impairs the remission of symptoms^7^, occupational and social functioning^7,8^, life satisfaction and wellbeing^9,10^, and the therapeutic alliance and psychological recovery as well^11^. The variability seen in FTD has frequently been depicted as a dichotomy between negative and positive FTD, encompassing a quantitative deficiency in speech and thought production on one end and heightened speech and thought production on the other^2^. While the distinction between negative and positive FTD is well established^2,12^, factor analytic studies suggested up to five FTD factors explaining more variance than the negative-positive dichotomy^3,13,14^.

Importantly, the dimensions of FTD have clinical significance^3^. Positive and negative FTD are linked to different neuropsychological deficits^15^ across multiple neurocognitive domains^16–18^. Additionally, the negative FTD dimension more accurately predicted the progression to schizophrenia in individuals at risk for psychosis, irrespective of genetic risk^6^. Similarly, distinct dimensions of FTD (either positive or negative) predicted poor outcomes, unlike general FTD^3,19,20^.

Although different FTD dimensions can be distinguished on the behavioral level, the underlying neuropathologies are still a matter of debate. In fact, most of the studies focused on general FTD as a single construct and did not differentiate between different FTD dimensions. In detail, several studies linked general FTD severity to reduction of gray matter (GM) volume in frontal language-related regions^21–23^ (inferior frontal gyrus (IFG), Broca’s area, frontal operculum, and prefrontal cortex (PFC)) with some conflicting results^24–28^. Likewise temporal and parietal regions within the language network have been associated with FTD severity. Specifically, reductions in GM volume have been reported in the superior temporal gyrus (STG), including the planum temporale and temporal pole, either unilaterally (left)^21,24,25,29,30^ or bilaterally^26,31–35^, left superior temporal sulcus (STS)^24,25^, and left angular gyrus (AngG)^25^. In contrast, GM volume increase within the left^22^ and right^23,36^ STG was associated with FTD severity. Moreover, FTD severity has been linked to reduced or reversed asymmetries of the planum temporale^37,38^ again with some conflicting results^39^. However, other investigations have failed to identify significant associations between FTD severity and GM volumes of the STG^28,36,39–44^ and inferior parietal lobule^45^.

Of note, FTD has also been associated with GM volume reductions in cortical regions beyond the traditional language network such as the middle (MFG), medial, and superior frontal gyri (SFG)^33^, ventromedial PFC^21^, orbitofrontal cortex (OFC)^21,24,46^, precuneus^25,33^, cuneus/lingual gyrus (LingG)^24^, cingulate gyrus^25,33^, and the insula^28,33^. Moreover, alterations in the volumes of the parahippocampal gyrus^31^ and both reductions and increases in cerebellar volumes^47,48^ have been found to be significantly linked to FTD. Finally, investigations into GM correlates of FTD in subcortical areas have yielded inconsistent findings. Studies have reported varied results for the hippocampus-amygdala complex^26,31,34,43,49–51^, basal ganglia^27,33^, and no association was found between FTD and GM volume of the thalamus^52^. In essence, these studies reported GM volume deficits in the fronto-temporal language network as associated with FTD in schizophrenia^24,25,53^. In addition, the language network is closely tied to other important brain networks, such as the default mode, salience, and central executive networks and the identified regions, i.e., lateral and medial PFC and parietal, cingulate, and insular cortex, may at least partly reflect closely related processes such as memory, unification, and cognitive control^54^.

Hitherto only a small number of studies directly investigated GM correlates of FTD dimensions suggesting distinct pathophysiology of FTD dimensions^1,55,56^. A recent study based on a large multi-site cohort through the ENIGMA Schizophrenia Working Group (752 individuals with schizophrenia and 1,256 controls) investigated the neuroanatomy of positive, negative, and general FTD in schizophrenia^55^. Likewise, Maderthaner and colleagues investigated structural correlates (cortical thickness (CT) and GM volume) of four different FTD dimensions. In sum, while positive FTD was associated with altered structural correlates such as CT or GM volume of core language areas, i.e., MTG, STG, and IFG as well as with non-language-related areas, negative FTD is probably less clearly associated with language-related areas but with brain areas relevant for higher order control processes, i.e., MFG, anterior cingulate cortex (ACC), and PFC regions. These two studies^1,55^ suggest differential structural brain correlates for different FTD dimensions and show that non-language-related regions also contribute to FTD in schizophrenia. White matter dysconnectivity associated with the FTD dimensions disorganization and emptiness has also been reported^57^, mainly for cortical brain regions previously associated with FTD in schizophrenia^53,58^.

Similarly, a limited number of functional neuroimaging studies have directly explored the correlates of FTD dimensions, again indicating distinct pathophysiological mechanisms^59–62^. While positive FTD was linked to changes in task-based functional activation of the STG (Wernicke’s area), IFG, and the parahippocampal gyrus^59,60,62^ and to perfusion in the supplementary motor area and IFG^61^, negative FTD involved task-based functional activations in brain regions important for higher-order control processes such as the parietal lobe, cuneus, precuneus, and posterior frontal lobe^63,64^ but also altered perfusion in the STG^61^. Therefore, negative FTD may reflect impaired access to semantic memory, while positive FTD likely indicates an inability to suppress irrelevant information during increased speech production^61^. The severity of alterations in the language dimension was also associated with altered perfusion in the right Heschl’s gyrus, but perfusion did not differentiate between positive and negative FTD dimensions^65^.

Notable previous structural neuroimaging studies reviewed elsewhere^53,58^ focused mostly on GM volume only as a structural marker. However, to capture the exact nature of the morphological alterations in FTD dimensions, it is highly recommended to investigate multiple morphological measures. Importantly, voxel-based morphometry studies rely on GM volume as an anatomical marker forming a product of cortical surface area (CSA) and CT. Therefore, if these variables run in opposite directions, measurements can be confounded which may hamper to detect specific effects of FTD. In fact, CSA and CT are independent neuroanatomical traits influenced by different factors during brain development (e.g., CT and CSA appear to be modulated by different genes)^66^. While CSA increases during late fetal development due to cortical folding, CT alters dynamically across the entire life span because of training, experience, and disease^66^. Likewise, gyrification is an anatomical trait influenced by early brain development. Thus, CT, CSA, and gyrification are considered as independent markers best measured separately when investigating the neuroarchitecture of the human cortex^67^. However, to the best of our knowledge, altered gyrification in association with FTD has not been investigated yet. In fact, FTD occurs early in the course of schizophrenia^4–6^ pointing to alterations in early brain development. Thus, changes in neuromorphological markers related to early brain development such as gyrification and CSA^68,69^ may be particularly prominent in patients with FTD compared to changes in neuromorphological markers subjected to lifelong plastic processes such as CT^66^.

In the present study, we therefore investigated morphological correlates of FTD dimensions (positive, negative, and linguistic control) in schizophrenia patients using multiple cortical measures (CSA, CT, cortical volume (CV), as well as gyrification) based on surface-based morphometry. We tested whether FTD dimensions were associated with different structural brain correlates and hypothesized to detect prominent altered markers of brain maturation (gyrification, CSA). Furthermore, morphological correlates were expected to be evident rather in language-related brain regions for the positive and linguistic control FTD dimensions and in non-language-related brain regions for the negative FTD dimension.

## Methods

### Participants

The current study initially enrolled 93 patients with schizophrenia spectrum disorders according to Diagnostic and Statistical Manual of Mental Disorders (DSM-5) criteria. Patients were recruited from the in- and outpatient clinics at the University Hospital of Psychiatry and Psychotherapy in Bern, Switzerland. All patients were between 20 and 65 years old (Supplementary Table S1) and provided written informed consent to participate in the study. The study protocols adhered to the declaration of Helsinki and were approved by the local ethics committee.

General exclusion criteria for all participants were substance abuse/dependence other than nicotine, a history of neurologic disease, head trauma with concurrent loss of consciousness, electroconvulsive treatment, and any magnetic resonance imaging (MRI) counter-indication. Current symptom severity was assessed with the Positive and Negative Syndrome Scale^70^. All participants completed the Mini International Neuropsychiatric Interview^71^.

Twenty-three participants (three due to unclear diagnosis, one due to substance abuse, two due to missing clinical values, and 17 due to ringing artefacts or reduced quality of the MRI scans) were excluded resulting in a final sample size of 70 patients with schizophrenia spectrum disorders (51 patients with schizophrenia, 10 with schizoaffective disorder, and 9 with schizophreniform disorder). The demographic and clinical characteristics of the final sample are presented in Table 1.

**Table 1:** Demographic and clinical characteristics of the schizophrenia patients.

	Schizophrenia patients (*n* = 70)				
	Mean	Standard deviation	Minimum	Maximum	Number (n)
Age (years)	36.93	12.78	20.0	65.0	70
Education (years)	13.78	2.97	9.0	23.0	69
DOI (years)	8.46	8.45	0.5	36.5	69
OLZ eq. (incl. unmedi.)	12.35	8.60	0.0	32.9	70
OLZ eq. (excl. unmedi.)	13.73	7.95	2.4	32.9	63
PANSS positive	18.19	5.69	7.0	31.0	70
PANSS negative	16.37	5.06	7.0	31.0	70
PANSS general	33.99	7.78	18.0	55.0	70
PANSS total	68.69	14.70	41.0	108.0	70
FTD general (TLC sum)	7.63	8.83	0.0	38.0	70
FTD positive	0.56	0.69	0.0	2.6	70
FTD negative	0.31	0.52	0.0	2.0	70
FTD linguistic control	0.29	0.58	0.0	3.0	70
	Frequency (n / %)		Frequency (n / %)		
Sex (male, female)	44 / 62.9 (male)		26 / 37.1 (female)		70
FTD general (no, yes)	14 / 20.0 (no)		56 / 80.0 (yes)		70
FTD positive (no, yes)	21 / 30.0 (no)		49 / 70.0 (yes)		70
FTD negative (no, yes)	42 / 60.0 (no)		28 / 40.0 (yes)		70
FTD linguistic control (no, yes)	47 / 67.1 (no)		23 / 32.9 (yes)		70

Shown are the mean, standard deviation, minimum, maximum, and number and percent of subjects for the demographic and clinical variables of the final sample.

*%* percent, *DOI* duration of illness, *excl.* exclusive, *FTD* formal thought disorder, *incl.* inclusive, *n* number of participants, *OLZ eq.* olanzapine equivalents; *PANSS*, positive and negative syndrome scale, *TLC* thought, language, and communication scale, *unmedi.* unmedicated.

### Thought, language, and communication scale for measuring formal thought disorder dimensions

FTD dimensions were measured using the thought, language, and communication (TLC) scale^72^ according to a previous study of our group^61^. The FTD dimensions are based on a factor analysis published elsewhere^14^. The positive FTD dimension, also termed disorganization, was defined by the mean of the following ten items (derailment, loss of goal, circumstantiality, pressure of speech, tangentiality, distractible speech, self-reference, perseveration, incoherence, and stilted speech). The negative FTD dimension, also termed emptiness, was defined by the mean of the following three items (poverty of speech, blocking, and poverty of content), whereas the linguistic control FTD dimension was defined by the mean of the following three items (neologism, illogicality, and word approximations). Nagels and colleagues named this factor ‘linguistic control’ “since changes on the word basis were most characteristic”^14^. Phonemic and semantic paraphasia items were not included in the linguistic control FTD dimension because these two items were not part of the TLC scale. In addition, we also used the sum of all 18 TLC items to assess the severity of general FTD.

### Magnetic resonance imaging data acquisition

MRI was performed with two different machines: a 3 Tesla Siemens MAGNETOM Prisma and a 3 Tesla MAGNETOM Trio Tim scanner (Siemens Healthineers, Erlangen, Germany), both equipped with a 20-channel radio frequency head coil array and located at the University Hospital of Bern, Switzerland. On both scanners, identical pulse sequences with the same parameters have been applied. A 3D-T1-weighted magnetization prepared 2 rapid acquisition gradient echoes pulse sequence has been applied, providing 176 sagittal slices with 240 × 256 matrix points and a field of view of 240 × 256 mm^2^, yielding an isotropic voxel resolution of 1 mm^3^. Further scan parameters for this anatomical scan were 5,000 ms repetition time, 2.98 ms echo time, 700 and 2500 ms inversion time 1 and 2, respectively, and a flip angle 1 and 2 of α = 4 and 5°, respectively. Siemens’s integrated parallel acquisition technique generalized autocalibrating partial parallel acquisition with an acceleration factor 3 for phase encoding has been applied. Total acquisition time was 8 minutes 22 seconds.

### Imaging data processing

Cortical surface reconstruction and volumetric segmentation was performed with the FreeSurfer image analysis suite (version 7.1.1, https://surfer.nmr.mgh.harvard.edu/fswiki). Technical details of these procedures are described elsewhere^73–80^. FreeSurfer is designed to reconstruct surface-based morphological representations of different cortical measures such as CT, CSA, and CV that can be analyzed in isolation. The processing stream followed FreeSurfer’s standard procedure, and its single steps are described in detail in the Supplementary Methods. We analyzed vertex-wise CT, CSA, and CV as well as the local gyrification index (lGI) as implemented in FreeSurfer (https://surfer.nmr.mgh.harvard.edu/fswiki/LGI). The lGI is a metric that quantifies the amount of cortex buried within the sulcal folds as compared with the amount of cortex on the outer visible cortex. A cortex with extensive folding has a large lGI, whereas a cortex with limited folding has a small lGI^81^. FreeSurfer also provided the global measures mean CT, total white matter CSA, and total CV.

### Statistical analyses

#### Demographic and clinical variables

Descriptive statistics and correlations of the demographic and clinical variables were computed using IBM SPSS Statistics software (version 27, https://www.ibm.com/products/spss-statistics). Correlations of the TLC scores among the different FTD dimensions and between different FTD dimensions and the covariates of no interest were computed using Pearson’s correlation.

#### Vertex-wise analyses of the different surface-based morphological measures

The different surface-based morphological measures (CT, CSA, CV, and lGI) were analyzed using FreeSurfer’s implemented statistics tools (mri_glmfit). Partial correlations were computed with either the severity of the different FTD dimensions (positive, negative, and linguistic control) or the severity of general FTD (sum of all TLC items) as independent variable and the vertex-wise cortical measures as dependent variables while simultaneously controlling for sex, age, scanner, and corresponding global brain measures.

When calculating brain-behavior associations for the three distinct FTD dimensions, we also corrected for the other FTD dimensions. Due to the strong correlation between the positive and linguistic control FTD dimensions, we used only one FTD dimension that is not correlated with the FTD dimension of interest. Hence, the negative FTD dimension is used as a covariate when investigating the positive and linguistic control FTD dimensions and the positive FTD dimension is used as a covariate when investigating the negative FTD dimension. For CT and CV analyses, mean global CT and total global CV, respectively, were used as global brain covariates, whereas for CSA and lGI analyses total global white matter CSA was used as global brain covariate. These global cortical measures were used as covariates to account for the dependency between the local cortical measures and the global ones and to increase regional specificity of the findings^66,82,83^. To rule out any influence of medication, we run also analyses additionally controlling for medication (olanzapine equivalents calculated according to Leucht and colleagues^41^). Vertex-wise analyses were also corrected for multiple comparisons using Monte Carlo simulations (10,000 repetitions) of the cluster size (mri_glmfit-sim with -mczsim option). Cluster-wise p-value was set at *p* < 0.05 corrected.

## Results

### Demographic and clinical characteristics

Demographic and clinical characteristics of the final sample (after exclusions, *n* = 70) are presented in Table 1. The characteristics of the initial sample (before exclusions, *n* = 93) are presented in Supplementary Table S1. Correlations among FTD dimensions and between different FTD dimensions and the covariates of no interest are reported in Supplementary Results and Table S2, respectively.

### Positive formal thought disorder dimension

**Fig. 1:**
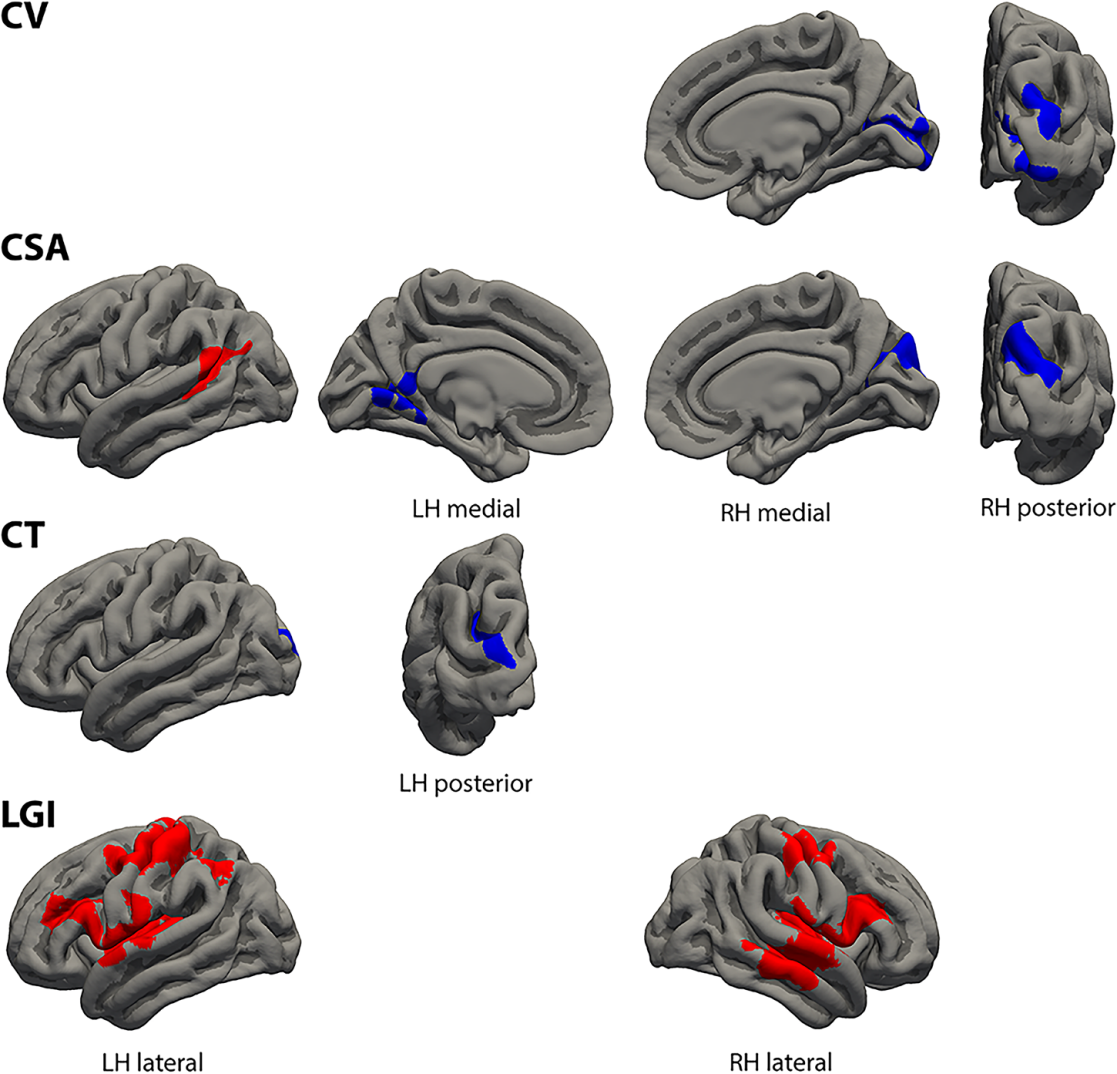
Clusters of different cortical morphological measures associated with the positive formal thought disorder dimension. Clusters represent partial correlations (positive in red, negative in blue) corrected for sex, age, negative formal thought disorder dimension, scanner, and corresponding global measures as well as corrected for multiple comparisons using Monte Carlo simulations of the cluster size. Cluster information is summarized in Table 2. CSA cortical surface area, CT cortical thickness, CV cortical volume, LGI local gyrification index, LH left hemisphere, RH right hemisphere.

The morphological cortical clusters associated with the severity of the positive FTD dimension are summarized in Table 2 and visualized in Fig. 1. Most prominent results were detected with the lGI. Positive FTD severity was positively correlated with the lGI of core (semantic) language areas including bilateral Heschl’s gyrus and STG as well as core language production areas (i.e., Broca’s area and its homologue). Positive FTD was also positively correlated with the lGI of bilateral insula, MFG, sub- (SubCG), pre- (PreCG), and postcentral gyrus (PostCG), and left AngG and supramarginal gyrus (SupraMG) as well as with CSA of Wernicke’s area. However, we also detected inverse associations of the positive FTD dimension with CV, CT, and/or CSA of brain areas that are not part of the language system (bilateral occipital cortex, left LingG, and right cuneus). Analyses with medication as additional covariate revealed substantially the same results (details of the clusters with medication as covariate see Supplementary Table S4).

**Table 2:** Clusters of different cortical morphological measures associated with the positive formal thought disorder dimension.

Max.	No. Vtx. Max.	Size (mm^2^)	MNI (X)	MNI (Y)	MNI (Z)	CWP	CWP (Low)	CWP (High)	No. Vtxs.	Weight Vtx.	Brain regions
Left cortical volume – No significant clusters											
**Right cortical volume**											
−4.160	28,459	1696	16	−94	−10	0.0001*	0	0.0002	2369	−4364	Cuneus, calcarine sulcus, occipital pole
−3.332	31,390	755	17	−88	19	0.0475	0.0448	0.0502	1000	−1902	Occipital pole, SOG
**Left cortical surface area**											
−2.578	31,675	1270	−8	−49	14	0.0031*	0.0024	0.0038	2732	−4742	LingG, calcarine sulcus, ventral PCC
4.558	18,380	1191	−60	−51	17	0.0047*	0.0038	0.0056	2626	6062	STS, planum temporale, SupraMG, AngG
**Right cortical surface area**											
−3.043	101,252	1695	16	−90	19	0.0001*	0	0.0002	2406	−4493	Occipital pole, SOG, cuneus
**Left cortical thickness**											
−2.823	9559	754	−30	−68	25	0.0249	0.0229	0.0269	1278	−2240	Occipital pole, MOG, SOS
Right cortical thickness – No significant clusters											
**Left local gyrification index**											
2.945	73,392	5522	−52	21	18	0.0001*	0	0.0002	12,768	23,527	STG, HG, insula, SubCG, CS, opercular/triangular IFG, IFS, MFG
2.804	54,841	5127	−39	4	44	0.0001*	0	0.0002	12,062	21,629	MFG, PreCG, CS, PostCG, PostCS, AngG, SupraMG
**Right local gyrification index**											
4.001	75,834	6227	51	−24	−13	0.0001*	0	0.0002	13,896	25,277	MTG, STS, STG, HG, insula, SubCG, opercular/triangular IFG, IFS
2.332	103,579	2165	40	−2	44	0.0001*	0	0.0002	4740	7699	MFG, PreCG, CS, PostCG, PostCS

Clusters represent partial correlations corrected for sex, age, negative formal thought disorder dimension, scanner, and corresponding global measures as well as corrected for multiple comparisons using Monte Carlo simulations of the cluster size. Clusters are visualized in Fig. 1.

*p-value surviving Bonferroni correction for the four different measures (*p* < 0.0125). *AngG* angular gyrus, *CWP* cluster-wise p-value, *CWP* Low and CWP High, 90% confidence interval for CWP, *CS* central sulcus, *HG* Heschl’s gyrus, *IFG* inferior frontal gyrus, *IFS* inferior frontal sulcus, *LingG* lingual gyrus, *Max.* maximum –log10(p*-*value) in the cluster (positive/negative values represent positive/negative correlations), *MFG* middle frontal gyrus, *MNI* (XYZ) the Montreal Neurological Institute coordinates of the maximum, *MOG* middle occipital gyrus, *MTG* middle temporal gyrus, *No. Vtx. Max.* vertex number at the maximum, *No. Vtxs.* number of cluster vertices, *PCC* posterior cingulate cortex, *PostCG* postcentral gyrus, *PostCS* postcentral sulcus, *PreCG* precentral gyrus, *Size* cluster size in mm^2^, *SOG* superior occipital gyrus, *SOS* superior occipital sulcus, *STG* superior temporal gyrus, *STS* superior temporal sulcus, *SubCG* subcentral gyrus, *SupraMG* supramarginal gyrus, *Vtx.* vertex, *Vtxs.* vertices, *Weight Vtx*. weight of cluster (size x intensity).

### Linguistic control formal thought disorder dimension

**Fig. 2:**
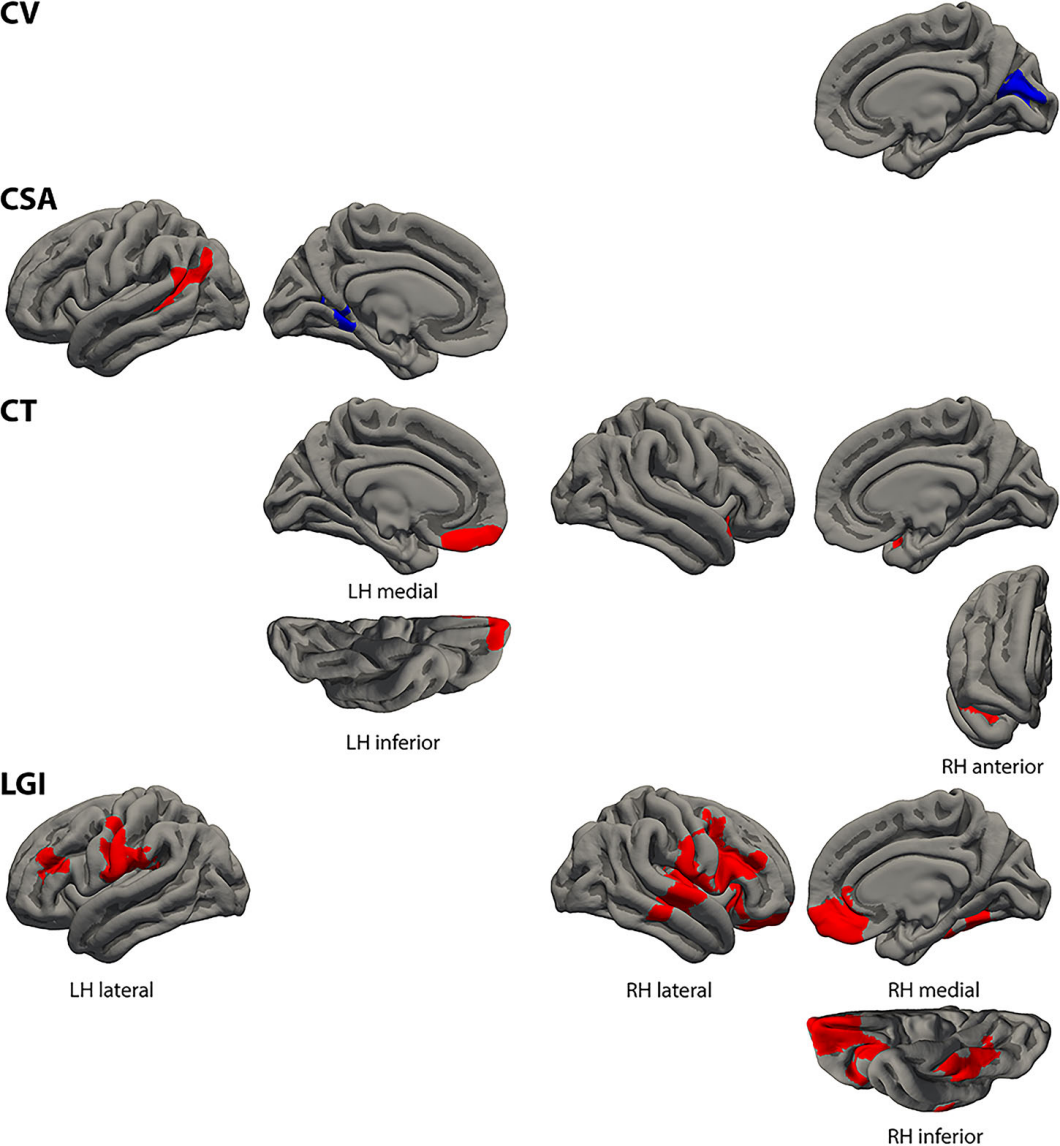
Clusters of different cortical morphological measures associated with the linguistic control formal thought disorder dimension. Clusters represent partial correlations (positive in red, negative in blue) corrected for sex, age, negative formal thought disorder dimension, scanner, and corresponding global measures as well as corrected for multiple comparisons using Monte Carlo simulations of the cluster size. Cluster information is summarized in Table 3. CSA cortical surface area, CT cortical thickness, CV cortical volume, LGI local gyrification index, LH left hemisphere, RH right hemisphere.

The morphological cortical clusters associated with the severity of the linguistic control FTD dimension are summarized in Table 3 and visualized in Fig. 2. There was a considerable overlap of the detected associations with those found for the positive FTD dimension. Specifically, again the lGI of core language areas (i.e., Broca’s area and its homologue, right MTG and STG) was positively associated with linguistic control FTD. In contrast to the positive FTD dimension we did not detect associations of the lGI and the left STG. Linguistic control FTD was also positively correlated with the lGI of bilateral MFG, SubCG, and PostCG, left PreCG and SupraMG, right insula, OFC, frontal pole, ventral ACC, fusiform gyrus, and LingG. As with positive FTD, linguistic control FTD was also positively correlated with CSA of Wernicke’s area. In addition, linguistic control FTD was inversely associated with CV and CSA of brain areas that are not part of the language system such as the bilateral occipital cortex, left LingG, and right cuneus as well as positively correlated with CT of the left OFC, right anterior STG, and insula. Again, analyzing associations with medication as additional covariate only marginally changed the results (details of the clusters with medication as covariate see Supplementary Table S5).

**Table 3:** Clusters of different cortical morphological measures associated with the linguistic control formal thought disorder dimension.

Max.	No. Vtx. Max.	Size (mm^2^)	MNI (X)	MNI (Y)	MNI (Z)	CWP	CWP (Low)	CWP (High)	No. Vtxs.	Weight Vtx.	Brain regions
Left cortical volume – No significant clusters											
**Right cortical volume**											
-2.634	61,400	1204	14	-79	13	0.0016*	0.0011	0.0021	1617	-2695	Cuneus, calcarine sulcus
**Left cortical surface area**											
3.993	147,035	1343	-58	-52	18	0.0020*	0.0014	0.0026	2901	6181	STS, SupraMG, AngG
-3.176	79,916	828	-16	-49	1	0.0485	0.0458	0.0513	1913	-3576	LingG, ventral PCC, POS
Right cortical surface area – No significant clusters											
**Left cortical thickness**											
4.719	6655	690	-10	53	-22	0.0429	0.0403	0.0455	1040	2368	OFC, rectal gyrus
**Right cortical thickness**											
3.107	4312	897	46	-3	-19	0.0085*	0.0073	0.0097	2129	4254	Planum polare, anterior STG, AIC
**Left local gyrification index**											
3.220	150,611	2,643	-63	-10	25	0.0001*	0	0.0002	6177	10,878	SubCG, PreCG, CS, PostCG, SupraMG
2.733	65,645	914	-41	35	25	0.0281	0.026	0.0302	1478	2500	Opercular/triangular IFG, IFS, MFG
**Right local gyrification index**											
3.817	53,801	6,284	41	40	24	0.0001*	0	0.0002	13,389	24,373	MTG, STS, STG, HG, insula, SubCG, PostCG, opercular/triangular IFG, IFS, MFG
3.621	118,094	3,284	20	17	-21	0.0001*	0	0.0002	6109	12,103	AIC, orbital IFG, OFC, rectal gyrus, frontal pole, ventral ACC
2.281	162,343	995	40	-35	-22	0.0169	0.0153	0.0186	1852	2916	Fusiform gyrus, LingG

Clusters represent partial correlations corrected for sex, age, negative formal thought disorder dimension, scanner, and corresponding global measures as well as corrected for multiple comparisons using Monte Carlo simulations of the cluster size. Clusters are visualized in Fig. 2.

Abbreviations: *, p-value surviving Bonferroni correction for the four different measures (p < 0.0125). *ACC* anterior cingulate cortex, *AIC*, anterior insular cortex, AngG, angular gyrus, *CWP*, cluster-wise p-value, CWP Low and CWP High, 90% confidence interval for CWP, *HG*, Heschl,s gyurs; *IFG*, inferior frontal gyrus, *IFS*, inferior frontal sulcus, LingG, lingual gyrus, Max., maximum –log10(p*-*value) in the cluster (positive/negative values represent positive/negative correlations), *MFG*, middle frontal gyrus, *MNI (XYZ)*, the Montreal Neurological Institute coordinates of the maximum, *MTG*, middle temporal gyrus; No. Vtx. Max., vertex number at the maximum, No. Vtxs., number of cluster vertices, *OFC*, orbitofrontal cortex, *POS*, parieto-occipital sulcus; *PostCG*, postcentral gyrus; *PreCG*, precentral gyrus; Size, cluster size in mm^2^, *STG*, superior temporal gyrus, *STS*, superior temporal sulcus, *SubCG*, subcentral gyrus, *SupraMG*, supramarginal gyrus, Vtx., vertex, Vtxs., vertices, Weight Vtx., weight of cluster (size x intensity).

### Negative formal thought disorder dimension

**Fig. 3:**
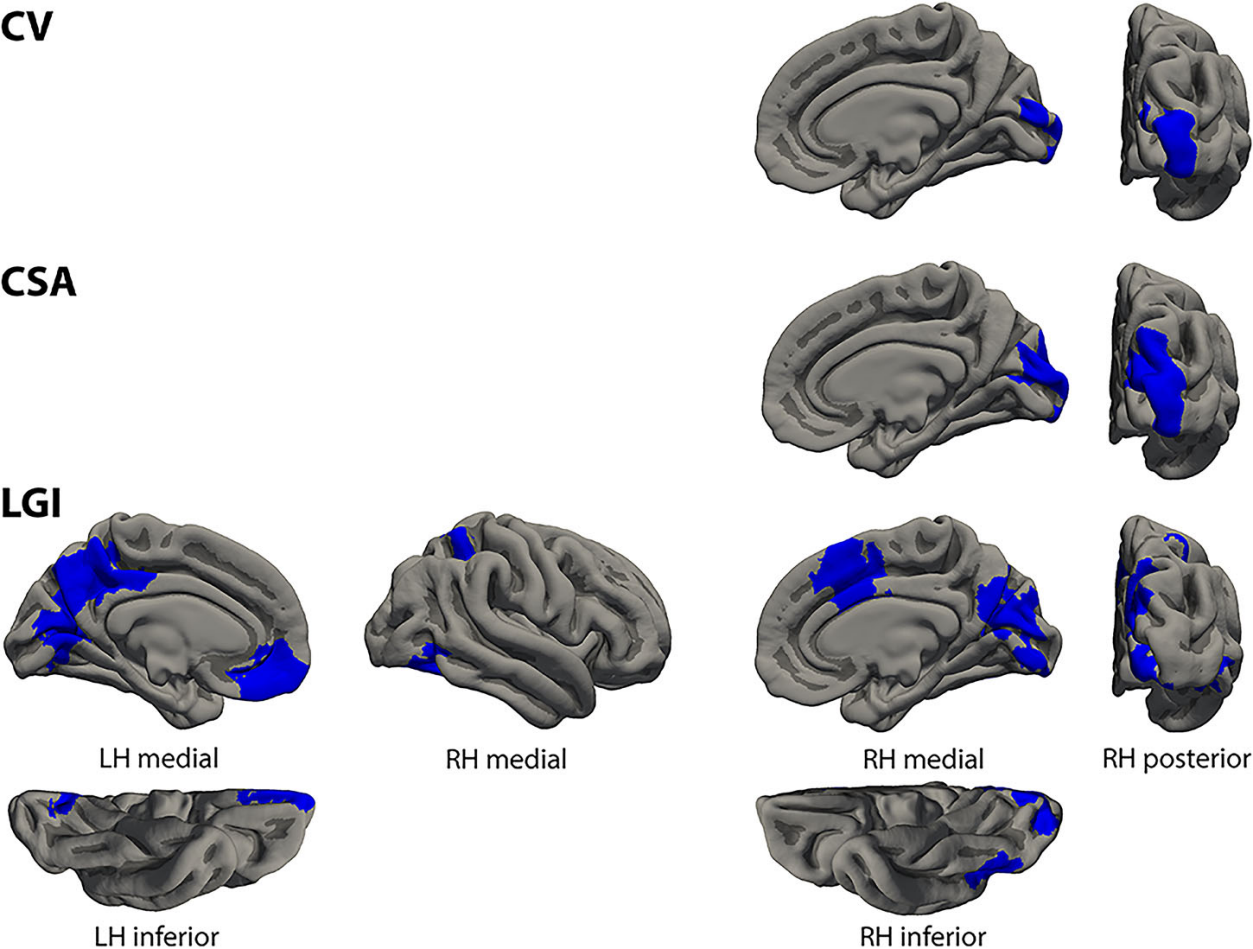
Clusters of different cortical morphological measures associated with the negative formal thought disorder dimension. Clusters represent partial correlations (negative in blue) corrected for sex, age, positive formal thought disorder dimension, scanner, and corresponding global measures as well as corrected for multiple comparisons using Monte Carlo simulations of the cluster size. Cluster information is summarized in Table 4. CSA cortical surface area, CV cortical volume, LGI local gyrification index, LH left hemisphere, RH right hemisphere.

The morphological cortical clusters associated with the severity of the negative FTD dimension are summarized in Table 4 and visualized in Fig. 3. In general, clusters found to be associated with the negative FTD dimension were different from those reported for the severity of the other FTD dimensions. Again, most prominent results were detected with the lGI. While we detected no associations of the negative FTD dimension with core language areas, we found inverse associations of the negative FTD dimension with lGI of bilateral LingG, occipital cortex, cuneus, and precuneus, left OFC and ventral ACC, right medial SFG, middle ACC, superior parietal lobule, intraparietal sulcus, fusiform gyrus, and inferior temporal sulcus. In addition, negative FTD was inversely correlated with CV and CSA of right occipital pole, cuneus, and superior occipital gyrus. As with the other factors associations with medication as additional covariate yielded substantially the same results (details of the clusters with medication as covariate see Supplementary Table S6).

**Table 4:** Clusters of different cortical morphological measures associated with the negative formal thought disorder dimension.

Max.	No. Vtx. Max.	Size (mm^2^)	MNI (X)	MNI (Y)	MNI (Z)	CWP	CWP (Low)	CWP (High)	No. Vtxs.	Weight Vtx.	Brain regions
Left cortical volume – No significant clusters											
**Right cortical volume**											
−3.491	129,181	1435	24	−98	−5	0.0003*	0.0001	0.0005	1811	−3641	Occipital pole, cuneus
Left cortical surface area – No significant clusters											
**Right cortical surface area**											
−4.657	125,808	2724	15	−99	12	0.0001*	0	0.0002	3539	−7219	Occipital pole, SOG, cuneus
Left cortical thickness – No significant clusters											
Right cortical thickness – No significant clusters											
**Left local gyrification index**											
-4.077	159,106	5610	−13	−51	40	0.0001*	0	0.0002	11,259	−20,583	LingG, calcarine sulcus, cuneus, POS, precuneus, dorsal PCC
−1.991	140,680	1415	−11	43	0	0.0013*	0.0009	0.0018	2375	−3638	OFC, rectal gyrus, ventral ACC
**Right local gyrification index**											
−3.150	104,309	4096	13	−63	27	0.0001*	0	0.0002	6269	−10,621	Occipital pole, LingG, calcarine sulcus, cuneus, POS, precuneus
−2.948	123,518	1667	13	12	40	0.0001*	0	0.0002	3755	−6521	Middle ACC, medial SFG
−2.007	56,460	992	33	−48	38	0.0172	0.0155	0.0189	2422	−3749	SPL, IPS
−2.374	144,063	983	40	−62	−13	0.0183	0.0166	0.02	1506	−2589	Fusiform gyrus, IOG, ITS

Clusters represent partial correlations corrected for sex, age, positive formal thought disorder dimension, scanner, and corresponding global measures as well as corrected for multiple comparisons using Monte Carlo simulations of the cluster size. Clusters are visualized in Fig. 3.

*p-value surviving Bonferroni correction for the four different measures (*p* < 0.0125). *ACC* anterior cingulate cortex, *CWP* cluster-wise p-value, *CWP* Low and CWP High, 90% confidence interval for CWP, *IOG* inferior occipital sulcus, *IPS* intraparietal sulcus, *ITS* inferior temporal sulcus, *LingG* lingual gyrus, *Max.* maximum –log10(p*-*value) in the cluster (positive/negative values represent positive/negative correlations), *MNI (XYZ)* the Montreal Neurological Institute coordinates of the maximum, *No. Vtx. Max.* vertex number at the maximum, *No. Vtxs.* number of cluster vertices, *OFC* orbitofrontal cortex, *PCC* posterior cingulate cortex, *POS* parieto-occipital sulcus, *Size* cluster size in mm^2^, *SFG* superior frontal gyrus, *SOG* superior occipital gyrus, *SPL* superior parietal lobule, *Vtx.* vertex, *Vtxs.* vertices, *Weight Vtx.* weight of cluster (size x intensity).

### Severity of general formal thought disorder

The morphological cortical clusters associated with the severity of general FTD are described in Supplementary Results, summarized in Supplementary Table S3, and visualized in Supplementary Fig. S1. In general, these clusters are very similar to the clusters found to be associated with the positive and partially linguistic control FTD dimensions reported above. The clusters found with medication as an additional covariate are also described in Supplementary Table S7).

## Discussion

FTDs form a core symptom dimension evident in multiple psychiatric conditions with unknown pathophysiology. Here we report associations of FTD dimensions with structural brain correlates of multiple morphological markers (CSA, CT, CV) and for the first time with lGI as a marker of early brain development. As hypothesized, we detected prominent altered markers of brain maturation (lGI) and replicated morphological correlates to be evident rather in language-related brain regions for the positive and linguistic control FTD dimensions and in non-language-related brain regions for the negative FTD dimension.

The main novelty are positive correlations between lGI and the severity of the positive and partially also the linguistic control FTD dimensions within fronto-temporal language network regions. Concurrently, we found inverse correlations between the severity of the negative FTD dimension and lGI in occipito-parietal and OFC regions. Thus, results suggest early neurodevelopmental alterations in language areas in subjects who later develop schizophrenia with predominant positive or linguistic control FTD.

Our study also replicated previous published findings of FTD dimension-specific morphological correlates within and beyond the language network. In the following, the associations with different markers investigated, i.e., markers associated with processes of early brain maturation (CSA, lGI) versus those rather associated with processes of neuroplasticity (CT) will be discussed first. We then discuss the different brain regions associated with the different FTD dimensions and how well these regions and their proposed brain functions fit with the literature. Finally we discuss whether our findings support the “dyssemantic hypothesis“^84^ or rather the “dysexecutive hypothesis“^85^ of FTD in schizophrenia.

### Association with markers influenced by brain development (lGI, CSA) and plasticity (CV, CT), respectively

Remarkably, investigating multiple morphological measures of FTD dimensions revealed distinguishable patterns of brain regions as associated with different markers. In fact, most prominent findings were shown with lGI. Notably, severity of positive and linguistic control FTD was associated with morphological markers in fronto-temporal language regions that are influenced by various factors during brain development (lGI). However, effects were not shown in these brain regions with morphological markers of plasticity changes during the lifespan (CV, CT). Likewise, for negative FTD most prominent findings were present for lGI. Yet findings of CSA only partly support this notion. Still, we may speculate that brain alterations in lGI associated with FTD may at least partly happen already early during (prenatal) brain development^68,69^ with regional differences, i.e., in fronto-temporal language regions for positive FTD and in occipito-parietal regions for negative FTD. This fits to the clinical observation that FTD can occur early in the course of the disease^4–6^. White and colleagues reviewed several prenatal and early postnatal events (symmetric and asymmetric cell division) that provide a critical foundation for subsequent pathology in gyrification during childhood, adolescence, and into adulthood^68^. Several factors may impact developmental precursors that contribute to gyrification. Interestingly genetic processes are suggested to play a large role in gyrification, especially early in development^68^. Kircher and colleagues highlight volume reductions in parts of language-related Wernicke’s and Broca’s areas as a highly consistent finding in structural MRI meta-analyses and mega-analyses in patients with schizophrenia and therefore suggest these alterations as probably of neurodevelopmental, and in particular of genetic, origin^2^. They suggest risk genes involved in the glutamatergic system led to dysfunctional glutamatergic neurotransmission and synaptic rarefication in the STG as a pathogenic route to positive FTD. Our results support this notion and further build on previous reports and theoretical models applying multiple morphological measures and specifically for the first time lGI measures. However, evidence suggest that general environmental risk factors for schizophrenia (neurodevelopmental, childhood abuse, migration, cannabis, etc.) also contribute to the pathophysiology of FTD in schizophrenia (for review see ref. ^2^). Furthermore, findings point to the importance to investigate multiple morphological markers to capture the full picture of alterations as associated with FTD dimensions. From a methodological point of view that is of specific relevance if measures go in different directions, which could hinder to detect changes in GM volume measured with voxel-wise morphometric procedures.

### Fronto-temporal language network

Here we report that the severity of the general, positive, and partially also of the linguistic control FTD dimensions but not the severity of the negative FTD dimension was associated with morphological features such as lGI of fronto-temporal language regions. In detail, the severity of the positive FTD dimension was positively correlated with lGI in bilateral fronto-temporal regions encompassing Broca’s area and its homologous region but sparing Wernicke’s area and its homologous region. In addition, the severity of the positive FTD dimension was positively correlated with CSA in left temporo-parietal regions partially overlapping with Wernicke’s area. The fronto-temporal morphological correlates of the general and linguistic control FTD dimensions were very similar to those found for the positive FTD dimension. In contrast, no fronto-temporal correlates have been found for the negative FTD dimension. These results are generally in line with the literature on FTD independent of FTD dimensions. In fact, with respect to fronto-temporal language-related regions associated with FTD in general, GM volume decreases or inverse correlations^21–26,29–35^ as well as increases or positive correlations^22,23,33,36^ have repeatedly been reported as reviewed elsewhere^53,58^. Likewise, our results corroborate previous reports that concentrate on FTD dimensions with some discrepancy in the direction of effects^1,55,86^. While two studies and our study found similar temporal brain regions associated with positive FTD, in our study positive FTD severity was positively correlated with lGI whereas two studies^55,86^ reported inverse correlations with CT and/or CSA possibly related to the sample, measure of FTD dimension, morphological measure, and/or the covariates of no interest used in the statistical models.

In sum, our study back up previous studies that suggest the positive and linguistic control FTD dimensions as related to both language production (Broca’s area and its homologue) and perception (Wernicke’s area). In addition to these two classical language regions, cortical features of bilateral STG and MTG devoted to semantic processing^87^ underscored alterations in the functioning of the STG and MTG in FTD^88^, which are pivotal nodes within the human language processing network^89^. Thus, results support, the “dyssemantic hypothesis” of FTD^84^ for positive and linguistic control but not negative FTD dimensions (see below). Importantly, findings further suggest distinguishable changes pointing to distinguishable pathophysiology for FTD dimensions.

### Occipito-parietal and occipito-temporal regions

Both different as well as partially overlapping occipito-parietal and occipito-temporal regions were associated with the different FTD dimensions investigated. There were two partially overlapping clusters inversely associated with the severity of all FTD dimensions, albeit with different morphological features (CV, CSA, CT, and/or lGI). One cluster is located in the right cuneus and calcarine sulcus and the other cluster in the left LingG partially extending into the cuneus and calcarine sulcus. Previous studies already reported GM volume reductions of the right cuneus/LingG associated with enhanced FTD severity^24^, GM volume increases in the right LingG associated with the positive FTD dimension^1^, and positive correlations between general or positive FTD and CT in bilateral pericalcarine cortex, right cuneus, and left LingG as well as inverse correlations between positive FTD and CSA in bilateral LingG and pericalcarine cortex^55^. Although an involvement of occipito-parietal and occipito-temporal regions associated with FTD have been reported by some studies, these regions are commonly implicated in visual processing, and it remains unclear whether and how visual processes contribute to FTD. Importantly, occipito-temporal regions were unique for the negative FTD dimension and occipito-parietal correlates were more extensive for the negative compared to the other FTD dimensions. Because FTD typically involves disruptions in the organization, coherence, and logical structure of thought processes, visual information processing might indirectly influence cognitive functions through connections with other brain regions possibly most relevant for the pathophysiology of negative FTD. For example, abnormalities in occipital regions may affect higher-order cognitive processes indirectly linked to FTD, such as attention, memory, and language comprehension.

### Orbitofrontal regions

The linguistic control but not the positive FTD dimensions were positively correlated with CT of the left and with lGI of the right OFC. In addition, the negative FTD dimension was negatively correlated with lGI of the left OFC. OFC regions associated with FTD have also been reported by other studies^1,21,55^. The OFC is associated with cognitive and behavioral control^90,91^ and with affective processing^92^, and it has been reported that the OFC is also affected in schizophrenia in general^93^. Aberrations in OFC morphology could impair cognitive, behavioral, and affective control that indirectly influence thought and language processing and hence contribute to FTD.

### Other regions

The present study also found correlates of FTD dimensions in brain regions neither related to primary language nor to other cognitive functions. General, positive, and linguistic control FTD dimensions were positively correlated with lGI in bilateral PreCG and PostCG that are involved in motor and somatosensory processing. Although these regions have been reported to be affected in task-based and resting-state functional^59,94,95^ and structural^55^ neuroimaging studies of FTD, it remains unclear how these regions are related to FTD.

### Is formal thought disorder related to semantic or executive dysfunctioning?

There has been a prolonged debate regarding whether FTD arises due to impairments in the language processing network, as posited by the “dyssemantic hypothesis“^84^, or instead stems from deficiencies in higher-order cognitive functions, as proposed by the “dysexecutive hypothesis“^85^. It has also been proposed that the default mode, salience, and central executive network that interact with the language network might contribute to linguistic disorganization and impoverishment in schizophrenia^54^. Our findings provide some evidence for the “dyssemantic hypothesis” for the positive and linguistic control FTD dimension but not for the negative FTD dimension because morphological correlates in language-related regions were found only for the positive and linguistic control FTD dimensions but not for the negative FTD dimension. With respect to the “dysexecutive hypothesis”, less evidence is provided by our study because morphological correlates in the classical PFC regions associated with cognitive control and executive functioning^96^ were rather sparse and limited to the OFC and/or small spots in the lateral and medial PFC and ACC in the positive, linguistic control, and negative FTD dimensions. Hence, our findings suggest that the positive and linguistic control FTD dimensions might result from semantic as well as executive dysfunctions, whereas the pathophysiology of the negative FTD dimension might partially depend on executive dysfunctions and aberration of other than semantic functions. However, due to overlapping morphological correlates in occipital regions among all FTD dimensions, other than semantic and executive dysfunctions might also be involved in all FTD dimensions. What kind of additional mechanism might contribute to FTD is difficult to infer solely from the morphological occipito-parietal and occipito-temporal correlates found in the present study and further research is needed to link these structural alterations to FTD in schizophrenia.

### Overlap of clusters across formal thought disorder dimensions and morphological measures

As expected, due to the correlations of the TLC scores among the general, positive, and linguistic control FTD dimensions, there is a considerable overlap of the morphological clusters associated with these FTD dimensions which may indicate shared neurobiological changes associated with these FTD dimensions. In addition to these FTD dimension correlations, there are also correlations among the morphological measures (e.g., CV, CT, and CSA). However, CV is the product of CT times CSA, and we investigated all three measures which at least partly explains the overlap among the morphological clusters found.

## Limitations

There are several limitations of the current study worth mentioning. First, most of our patients were medicated, which, in principle, may influence brain structure^97^. Still, included OLZ equivalence dosage as additional covariate of no interest in our analyses yielded substantially the same results. Second, due to the correlational nature of our study, causality cannot be established. Third, a larger sample size would be needed to confirm the suggested FTD dimensions^14^ by confirmatory factor analysis^98^. Forth, due to the dependency of the morphological measures (CV, CT, and CSA) as well as the correlations among FTD dimensions (general, positive, and linguistic control FTD), the clusters found are not fully independent and therefore overlap among morphological measures and FTD dimensions. Last, although the number of items used to define the negative and linguistic control FTD dimension is rather low (three items each) and might therefore limit the validity and reliability of these two FTD dimensions, the FTD dimensions were measured using the well-established TLC scale^72^ and based on a factor analysis^14^.

## Conclusions

Our findings (I) implies that a marker of early brain development, i.e., local gyrification, might best capture the neuromorphological underpinnings of different FTD dimensions in schizophrenia, (II) support the heterogeneity of FTD in schizophrenia, (III) show FTD dimension-specific neuromorphological correlates, (IV) suggest a distinct pathophysiology at least for the negative compared to other FTD dimensions, (V) reveal that non-language-related brain regions might also contribute to FTD, and (VI) show that neuromorphological correlates are dependent on the morphological marker assessed suggesting that different markers should be investigated.

Importantly, investigating multiple morphological measures reveal distinguishable patterns of brain regions as associated with different markers for different FTD dimensions. In particular, the marker local gyrification associated with early brain development seems to be most sensitive of altered language-related brain regions of the positive and linguistic control FTD dimensions. Our results further reveal that on the one hand compared to the negative FTD dimension the positive and linguistic control FTD dimension are associated with different morphological features (CSA, CT, and/or local gyrification) in language-related (fronto-temporal cortex) as well as non-language-related (mainly OFC and occipital cortex) brain regions. On the other hand, our results also show that the negative FTD dimension is associated with different as well as partially overlapping non-language-related structural brain correlates (mainly occipito-parietal and occipito-temporal cortex) compared to the positive and linguistic control FTD dimensions. Finally, our study provides evidence that the positive and linguistic control FTD dimensions seem to be compatible with the “dyssemantic hypothesis“^84^ as well as “dysexecutive hypothesis“^85^ of FTD, whereas the negative FTD dimension is only partially compatible with the “dysexecutive hypothesis“^85^ but not with the “dyssemantic hypothesis“^84^ of FTD.

## Supplementary information

Supplemental material

## Data Availability

The statistical maps that support the findings of this study are available from the corresponding author upon reasonable request. The data are not publicly available due to containing information that could compromise research participant privacy or consent. Explicit consent to deposit raw imaging data was not obtained from the participants.
