# Supplementary material for: Differential structural cortical correlates of positive, negative, and linguistic control formal thought disorder dimensions in schizophrenia
Source: Schizophrenia (Heidelb). 2025 Jul 16;11(1):99. doi: 10.1038/s41537-025-00644-8 (PMC12267446; doi:10.1038/s41537-025-00644-8)
Supplement: Supplementary file 1 — Supplemental material [file 41537_2025_644_MOESM1_ESM.docx]

**Differential structural cortical correlates of positive, negative, and linguistic control formal thought disorder dimensions in schizophrenia**

Jürgen Hänggi^1*^, Sebastian Walther^1,2,3^, Nicole Gangl^1^, Frauke Conring^1^, Katharina Stegmayer^1,2^

**Affiliations**

^1^ Translational Research Center, University Hospital of Psychiatry and Psychotherapy, University of Bern, Bern, Switzerland; ^2^ Translational Imaging Center, Swiss Institute for Translational and Entrepreneurial Medicine, Bern, Switzerland; ^3^ Department of Psychiatry, Psychosomatics, and Psychotherapy, Center for Mental Health, University of Würzburg, Germany

^*^ Corresponding author

**Content of Supplementary**

1. **Supplementary Methods**

1.1. Imaging data processing

1. **Supplementary Results**

2.1. Clinical characteristics

2.2. Correlations between formal thought disorder dimensions and covariates of no interest

- 1. Positive formal thought disorder dimension
  2. Linguistic control formal thought disorder dimension
  3. Negative formal thought disorder dimension
  4. General formal thought disorder
  5. Overlap of clusters across formal thought disorder dimensions and morphological measures

**3. Supplementary Tables and Figures**

3.1. Supplementary Table S1

3.2. Supplementary Table S2

3.3. Supplementary Table S3

3.4. Supplementary Table S4

3.5. Supplementary Table S5

3.6. Supplementary Table S6

3.7. Supplementary Table S7

3.8. Supplementary Figure S1

**4. Supplementary References**

**1. Supplementary Methods**

**1.1. Imaging data processing**

FreeSurfer processing is a fully automated procedure involving segmentation of the cortical white matter (WM)^1^, tessellation of the gray/WM junction, inflation of the folded surface tessellation patterns^2^ and automatic correction of topological defects in the resulting manifold^3^. This surface was then used as the starting point for a deformable surface algorithm designed to find the gray/white and pial (gray matter/cerebrospinal fluid) surfaces with sub-millimeter precision^4^. The procedures for measuring cortical thickness (CT) have been validated against histological analysis^5^ and manual measurements^6,7^. This method uses both intensity and continuity information from the surfaces in the deformation procedure in order to be able to interpolate surface locations for regions in which the magnetic resonance imaging (MRI) image is ambiguous^4^. For each subject, CT of the cortical ribbon was computed on a uniform grid (comprised by vertices) with 1 mm spacing across both cortical hemispheres, with the thickness being defined by the shortest distance between the gray/white and pial surface models. The thickness maps produced are not limited to the voxel resolution of the image and are thus sensitive to detecting sub-millimeter differences between groups^4^.

Thickness measures were mapped to the inflated surface of each participant's brain reconstruction, allowing visualization of data across the entire cortical surface (i.e., gyri and sulci) without being obscured by cortical folding. Each subject's reconstructed brain was then morphed to an average spherical surface representation that optimally aligned sulcal and gyral features across subjects^2^. This procedure provides accurate matching of morphologically homologous cortical locations among participants based on each individual's anatomy while minimizing metric distortions. This transform was used to map the thickness measurements into a common spherical coordinate system. Beside CT, FreeSurfer is able to compute surface-based maps of cortical surface area (CSA) and cortical volume (CV) as well.

The data were then smoothed on the surface tessellation using an iterative nearest-neighbor averaging procedure (equivalent to applying a two-dimensional Gaussian smoothing kernel along the cortical surface with a full-width-at-half-maximum of about 10 mm). In addition, we calculated global brain measures such as total cortical GM volume, total cortical white matter surface area, and mean CT.

**2. Supplementary Results**

**2.1. Clinical characteristics**

Most thought, language, and communication (TLC) scores used to define the different formal thought disorder (FTD) dimensions were highly correlated. Total TLC scores (general FTD) were significantly positively correlated with those of the positive (r=0.965, p<0.001) and linguistic control (r=0.850, p<0.001) but not with those of the negative (r=0.140, p=0.246) FTD dimension. TLC scores of the positive FTD dimension were significantly positively correlated with those of the linguistic control (r=0.765, p<0.001) but not with those of the negative (r=-0.067, p=0.583) FTD dimension, whereas the TLC scores of the linguistic control and negative FTD dimensions were also not related (r=0.003, p=0.983).

**2.2. Correlations between formal thought disorder dimensions and covariates of no interest**

Pearson’s correlation coefficients between the TLC scores of the different FTD dimensions and the covariates of no interest used in the statistical models of the morphometric analyses are summarized in Supplementary Table S2. Although most of the covariates of no interest are not significantly associated with the FTD dimensions, all these covariates (maybe except for medication) are significantly related to the local cortical morphological measures investigated.

**2.3.** **Positive formal thought disorder dimension**

The morphological cortical clusters associated with the severity of the positive FTD dimension are summarized in Table 2 and visually presented in Figure 1.

The severity of the positive FTD dimension was inversely correlated with CV in (I) a cluster in the right cuneus, calcarine sulcus, and occipital pole and (II) in a cluster in the right occipital pole and superior occipital gyrus (SOG). No significant clusters were found for left CV.

The severity of the positive FTD dimension was inversely correlated with CSA in (I) a cluster in the left lingual gyrus (LinG), calcarine sulcus, and ventral posterior cingulate cortex (PCC) and positively correlated with CSA in (II) a cluster in the left superior temporal sulcus (STS), planum temporale, supramarginal gyrus (SupraMG), and angular gyrus (AngG). In the right hemisphere, severity of the positive FTD dimension was inversely correlated with CSA in (III) a cluster in the right occipital pole, SOG, and cuneus. CSA cluster (III) is in close neighborhood and overlaps with CV cluster (II).

The severity of the positive FTD dimension was inversely correlated with CT in (I) a cluster in the left occipital pole, middle occipital gyrus (MOG), and superior occipital sulcus (SOS). CT cluster (I) is partially located in homologous regions compared to CV cluster (II) and CSA cluster (III). No significant clusters were found for right CT. The severity of the positive FTD dimension was positively correlated with lGI in (I) a large cluster distributed over the left superior temporal gyrus (STG), Heschl’s gyrus (HG), insula, subcentral gyrus (SubCG), central sulcus (CS), opercular/triangular inferior frontal gyrus (IFG), inferior frontal sulcus (IFS), and middle frontal gyrus (MFG) and in (II) a cluster distributed over the left MFG, precentral gyrus (PreCG), CS, postcentral gyrus (PostCG), postcentral sulcus (PostCS), AngG, and SupraMG. In the right hemisphere, the severity of the positive FTD dimension was positively correlated with lGI in (III) a large cluster distributed over the right MTG, STS, STG, HG, insula, SubCG, opercular/triangular IFG, and IFS and (IV) in a cluster distributed over the right MFG, PreCG, CS, PostCG, and PostCS. LGI clusters (I and III) as well as (II and IV) are partially located in homologous regions.

**2.4. Linguistic** **control formal thought disorder dimension**

The morphological cortical clusters associated with the severity of the linguistic control FTD dimension are summarized in Table 3 and visually presented in Figure 2.

The severity of the linguistic control FTD dimension was inversely correlated with CV in (I) a cluster in the right cuneus and calcarine sulcus. No significant clusters were found for left CV.

The severity of the linguistic control FTD dimension was positively correlated with CSA in (I) a cluster in the left STS, SupraMG, and AngG and positively correlated with CSA in (II) a cluster in the left LingG, ventral PCC, and parieto-occipital sulcus (POS). No significant clusters were found for right CSA.

The severity of the linguistic control FTD dimension was positively correlated with CT in (I) a cluster in the left orbitofrontal cortex (OFC) and rectal gyrus and (II) a cluster in the right planum polare, anterior STG, and anterior insular cortex (AIC).

The severity of the linguistic control FTD dimension was positively correlated with lGI in (I) a cluster distributed over the left SubCG, PreCG, CS, PostCG, and SupraMG and (II) in a cluster distributed over the left opercular/triangular IFG, IFS, and MFG. In the right hemisphere, linguistic control FTD dimension was positively correlated with lGI in (III) in a large cluster distributed over the right MTG, STS, STG, HG, insula, SubCG, PostCG, opercular/triangular IFG, IFS, and MFG, (IV) a cluster in the right OFC, rectal gyrus, orbital IFG, AIC, frontal pole, and ventral anterior cingulate cortex (ACC), and (V) in a cluster in the right fusiform gyrus and LingG. LGI clusters (I, II, and III) are partially located in homologous regions and lGI cluster (IV) is partially located in homologous regions compared to CT cluster (I).

**2.5.** **Negative formal thought disorder dimension**

The morphological cortical clusters associated with the severity of the negative FTD dimension are summarized in Table 4 and visually presented in Figure 3. In general, clusters found to be associated with the negative FTD dimension were, except two clusters (see section 2.7. below), different from the ones reported for the severity of general FTD and the other FTD dimensions.

The severity of the negative FTD dimension was inversely correlated with CV in (I) a cluster in the right occipital pole and cuneus. No significant clusters were found for left CV.

The severity of the negative FTD dimension was inversely correlated with CSA in (I) a cluster in the right occipital pole, cuneus, and SOG. CSA cluster (I) largely overlaps with CV cluster (I). No significant clusters were found for left CSA.

No significant left- and right-hemispheric CT clusters associated with the severity of the negative FTD dimension were found.

The severity of the negative FTD dimension was inversely correlated with lGI in (I) a cluster in the left LingG, calcarine sulcus, cuneus, POS, precuneus, and dorsal PCC, (II) a cluster in the left OFC, rectal gyrus, and ventral ACC. In the right hemisphere, the negative FTD dimension was inversely correlated with lGI in (III) a cluster in the right occipital pole, LingG, calcarine sulcus, cuneus, POS, and precuneus, (IV) in a clusters in the right middle ACC and medial superior frontal gyrus (SFG), (V) in a cluster in the right superior parietal lobule (SPL) and intraparietal sulcus (IPS), and (VI) in a cluster in the right fusiform gyrus, inferior occipital gyrus (IOG), and inferior temporal sulcus (ITS). LGI clusters (I and III) are partially located in homologous regions and lGI cluster (III) partially overlaps with CV cluster (I) and CSA cluster (I).

**2.6. General** **formal thought disorder**

The morphological cortical clusters associated with the severity of general FTD are summarized in Supplementary Table S3 and visually presented in Supplementary Figure S1. In general, these clusters are very similar to the clusters found to be associated with the positive and partially linguistic control FTD dimensions which are reported in the main manuscript.

The severity of general FTD was inversely correlated with cortical volume (CV) in (I) a cluster in the left cuneus, calcarine sulcus, and LingG, (II) a cluster in the right cuneus, calcarine sulcus, occipital pole, and POS, (III) in another cluster in the right occipital pole and SOG. The severity of general FTD was positively correlated with CV in (IV) a cluster in the left STS, planum temporale, and SupraMG. Clusters (I) and (II) are partially located in homologous regions.

The severity of a general FTD was positively correlated with CSA in (I) a cluster in the left STS, planum temporale, SupraMG, and AngG and inversely correlated with CSA in (II) a cluster in the left LingG and calcarine sulcus and in (III) a cluster in the right cuneus, SOG, and POS. CSA clusters (I, II, and III) considerably overlap with CV clusters (IV, I, and II), respectively.

The severity of a general FTD was positively correlated with CT in (I) a cluster in the left OFC and rectal gyrus.

The severity of a general FTD was positively correlated with local gyrification (lGI) in (I) a large cluster distributed over the left STG, Heschl’s gyrus (HG), insula, subcentral gyrus (SubCG), opercular/triangular IFG, inferior frontal sulcus (IFS), and MFG and in (II) a cluster distributed over the left MFG, PreCG, CS, PostCG, PostCS, SupraMG, and AngG.

In the right hemisphere, the severity of a general FTD was positively correlated with lGI in (III) a large cluster distributed over the right MTG, STS, STG, HG, insula, SubCG, PostCG, opercular/triangular IFG, IFS, and MFG, (IV) a cluster in the right PostCG, CS, PreCG, and MFG, and (V) in a cluster in the right OFC, rectal gyrus, and ventral ACC. LGI clusters (I and III) as well as (II and IV) are partially located in homologous regions and lGI cluster (V) is partially located in a homologous region as the CT cluster (I).

**2.7. Overlap of clusters across** **formal thought disorder dimensions and morphological measures**

The severity of the general, positive, as well as linguistic control FTD dimensions was positively correlated with CSA of the left STS, planum temporale (except for linguistic control FTD), SupraMG, and AngG and inversely correlated with CV of the right cuneus and calcarine sulcus.

The severity of the general, positive, as well as linguistic control FTD dimensions was positively correlated with lGI of the left SubCG, PreCG, CS, PostCG, PostCS, SupraMG, opercular/triangular IFG, IFS, and MFG and lGI of the left STG, HG, insula, and AngG was positively correlated with the severity of the general and positive FTD dimensions but not with that of the linguistic control FTD dimension. In the right hemisphere, the severity of the general, positive, as well as linguistic control FTD dimensions was positively correlated with lGI of the right MTG, STS, HG, insula, SubCG, PostCG, opercular/triangular IFG, IFS, and MFG, and lGI of the right OFC, rectal gyrus, and ventral ACC was positively correlated with the severity of the general and linguistic control FTD dimensions but not with that of the positive FTD dimension.

There were only two partially overlapping clusters associated with all FTD dimensions (general, positive, negative, and linguistic control). One cluster is located in the right cuneus and/or calcarine sulcus and this cluster is inversely correlated with CV and/or CSA in general FTD, with CV and/or CSA in the positive FTD dimension, with CV in the linguistic control FTD dimension, and with CV and/or CSA and/or lGI in the negative FTD dimension. The other cluster is located in the left LingG partially extending into the cuneus and calcarine sulcus and this cluster is inversely correlated with CV and CSA in the general FTD, with CSA in the positive and linguistic control FTD dimensions, and with lGI in the negative FTD dimension.

1. **Supplementary Tables and Figures**

**3.1. Supplementary Table S1. Demographic and clinical characteristics** **of** **the schizophrenia patients.** Shown are the mean, standard deviation, minimum, maximum, and number and percent of subjects for the demographic and clinical variables of the initial sample.

|  | Schizophrenia patients (n = 93) | | | | |
| --- | --- | --- | --- | --- | --- |
|  | Mean | Standard deviation | Minimum | Maximum | Number (n) |
| Age (years) | 38.16 | 12.86 | 20.0 | 65.0 | 93 |
| Education (years) | 13.50 | 2.87 | 8.0 | 23.0 | 91 |
| DOI (years) | 9.48 | 9.14 | 0.5 | 36.5 | 91 |
| OLZ eq. (incl. unmedi.) | 13.99 | 10.95 | 0.0 | 53.9 | 93 |
| OLZ eq. (excl. unmedi.) | 15.49 | 10.47 | 2.4 | 53.9 | 84 |
| PANSS positive | 18.40 | 5.98 | 7.0 | 33.0 | 90 |
| PANSS negative | 16.37 | 5.86 | 7.0 | 35.0 | 90 |
| PANSS general | 34.13 | 8.27 | 16.0 | 55.0 | 90 |
| PANSS total | 69.01 | 15.77 | 33.0 | 108.0 | 90 |
| FTD general (TLC sum) | 7.59 | 8.50 | 0.0 | 38.0 | 90 |
| FTD positive | 0.55 | 0.67 | 0.0 | 2.6 | 90 |
| FTD negative | 0.34 | 0.50 | 0.0 | 2.0 | 90 |
| FTD linguistic control | 0.28 | 0.55 | 0.0 | 3.0 | 90 |
|  | Frequency (n / %) | | Frequency (n / %) | |  |
| Sex (male, female) | 57 / 61.3 (male) | | 36 / 38.7 (female) | | 93 |
| FTD general (no, yes) | 17 / 18.9 (no) | | 73 / 81.1 (yes) | | 90 |
| FTD positive (no, yes) | 27 / 30.0 (no) | | 63 / 70.0 (yes) | | 90 |
| FTD negative (no, yes) | 50 / 55.6 (no) | | 40 / 44.4 (yes) | | 90 |
| FTD linguistic control (no, yes) | 60 / 66.7 (no) | | 30 / 33.3 (yes) | | 90 |

Abbreviations: %, percent; DOI, duration of illness; excl., exclusive; FTD, formal thought disorder; incl., inclusive; n, number of participants; OLZ eq., olanzapine equivalents; PANSS, positive and negative syndrome scale; TLC, thought, language, and communication scale; unmedi., unmedicated.

**3.2. Supplementary Table S2. Correlations between formal thought disorder dimensions and covariates of no interest.** Shown are Pearson’s correlation coefficients (p-value in brackets) between the TLC scores of the different FTD dimensions and the covariates of no interest used in the statistical models of the morphometric analyses. Significant correlations are printed in bold and statistical trends (0.05<p<0.10) are printed in italics.

|  | General FTD | Positive FTD | Negative FTD | Linguistic control FTD |
| --- | --- | --- | --- | --- |
| Age | **0.262 (0.028)** | 0.194 (0.107) | **0.273 (0.022)** | *0.224 (0.063)* |
| Sex | -0.183 (0.129) | -0.148 (0.22) | -0.029 (0.811) | *-0.232 (0.053)* |
| OLZ eq. (incl. unmedi.) | *0.227 (0.059)* | *0.209 (0.082)* | 0.036 (0.766) | *0.228 (0.057)* |
| Total CV | -0.051 (0.674) | -0.002 (0.987) | **-0.244 (0.042)** | -0.024 (0.846) |
| Total CSA | 0.002 (0.986) | 0.03 (0.808) | -0.151 (0.212) | 0.031 (0.797) |
| Mean CT | -0.139 (0.25) | -0.099 (0.414) | -0.186 (0.124) | -0.13 (0.282) |

Abbreviations: CSA, cortical white matter surface area; CT, cortical thickness; CV, cortical volume; FTD, formal thought disorder; incl., inclusive; OLZ eq., olanzapine equivalents; TLC, thought, language, and communication scale; unmedi., unmedicated.

**3.3. Supplementary Table S3. Clusters of different cortical morphological measures associated with general formal thought disorder.** Clusters represent partial correlations corrected for sex, age, scanner, and corresponding global measures as well as corrected for multiple comparisons using Monte Carlo simulations of the cluster size. Clusters are visualized in Supplementary Figure S1.

| **Max.** | **No. Vtx. Max.** | **Size (mm^2^)** | **MNI (X)** | **MNI (Y)** | **MNI (Z)** | **CWP** | **CWP (Low)** | **CWP (High)** | **No. Vtxs.** | **Weight Vtx.** | **Brain regions** |
| --- | --- | --- | --- | --- | --- | --- | --- | --- | --- | --- | --- |
| **Left cortical volume** | | | | | | | | | | | |
| -3.675 | 102,026 | 1,172 | -6 | -72 | 1 | 0.0027* | 0.002 | 0.0034 | 1,889 | -3,519 | Cuneus, calcarine sulcus, LingG |
| 4.458 | 1,914 | 782 | -60 | -51 | 19 | 0.0372 | 0.0348 | 0.0396 | 1,760 | 3,970 | STS, planum temporale, SupraMG |
| **Right cortical volume** | | | | | | | | | | | |
| -4.770 | 62,791 | 2,712 | 16 | -94 | -10 | 0.0001* | 0 | 0.0002 | 3,871 | -7,123 | Cuneus, calcarine sulcus, occipital pole, POS |
| -3.416 | 68,911 | 791 | 17 | -88 | 19 | 0.0353 | 0.0329 | 0.0377 | 1,045 | -2,046 | Occipital pole, SOG |
| **Left cortical surface area** | | | | | | | | | | | |
| 5.073 | 106,811 | 1,386 | -59 | -51 | 17 | 0.0016* | 0.0011 | 0.0021 | 2,991 | 7,218 | STS, planum temporale, SupraMG, AngG |
| -2.674 | 79,969 | 1,381 | -19 | -54 | -4 | 0.0017* | 0.0012 | 0.0022 | 2,958 | -5,361 | LingG, calcarine sulcus, ventral PCC |
| **Right cortical surface area** | | | | | | | | | | | |
| -3.303 | 46,011 | 1,780 | 16 | -90 | 19 | 0.0001* | 0 | 0.0002 | 2,521 | -4,842 | Cuneus, SOG, POS |
| **Left cortical thickness** | | | | | | | | | | | |
| 3.641 | 67,832 | 677 | -10 | 53 | -21 | 0.0476 | 0.0449 | 0.0503 | 1,069 | 2,171 | OFC, rectal gyrus |
| Right cortical thickness – No significant clusters | | | | | | | | | | | |
| **Left local gyrification index** | | | | | | | | | | | |
| 2.920 | 116,337 | 5,261 | -49 | -15 | 16 | 0.0001* | 0 | 0.0002 | 12,199 | 21,806 | STG, HG, insula, SubCG, opercular/triangular IFG, IFS, MFG |
| 2.336 | 99,857 | 3,441 | -44 | 2 | 43 | 0.0001* | 0 | 0.0002 | 8,089 | 13,234 | MFG, PreCG, CS, PostCG, PostCS, SupraMG, AngG |
| **Right local gyrification index** | | | | | | | | | | | |
| 3.979 | 140,741 | 7,207 | 46 | 16 | 20 | 0.0001* | 0 | 0.0002 | 15,823 | 28,715 | MTG, STS, STG, HG, insula, SubCG, PostCG, opercular/triangular IFG, IFS, MFG |
| 2.064 | 103,579 | 1,638 | 40 | -2 | 44 | 0.0001* | 0 | 0.0002 | 3,512 | 5,329 | PostCG, CS, PreCG, MFG |
| 2.198 | 24,994 | 1,109 | 18 | 46 | -15 | 0.0073* | 0.0062 | 0.0084 | 1,940 | 3,097 | OFC, rectal gyrus, ventral ACC |

Abbreviations: *, p-value surviving Bonferroni correction for the four different measures (p<0.0125). ACC, anterior cingulate cortex; AngG, angular gyrus; CS, central sulcus; CWP, cluster-wise p-value; CWP Low and CWP High, 90% confidence interval for CWP; Max., maximum –log10(p*-*value) in the cluster (positive/negative values represent positive/negative correlations); HG, Heschl’s gyrus; IFG, inferior frontal gyrus; IFS, inferior frontal sulcus; LingG, lingual gyrus; MFG, middle frontal gyrus; MNI (XYZ), the Montreal Neurological Institute coordinates of the maximum; MTG, middle temporal gyrus; No. Vtx. Max., vertex number at the maximum; No. Vtxs., number of cluster vertices; OFC, orbitofrontal cortex; PCC, posterior cingulate cortex; POS, parieto-occipital sulcus; PostCG, postcentral gyrus; PostCS, postcentral sulcus; PreCG, precentral gyrus; Size, cluster size in mm^2^; SOG, superior occipital gyrus; SubCG, subcentral gyrus; STG, superior temporal gyrus; STS, superior temporal sulcus; SupraMG, supramarginal gyrus; Vtx., vertex; Vtxs., vertices; Weight Vtx., weight of cluster (size x intensity).

**3.4. Supplementary Table S4. Clusters of different cortical morphological measures associated with the positive formal thought disorder dimension****.** Clusters represent partial correlations corrected for sex, age, negative formal thought disorder dimension, scanner, medication, and corresponding global measures as well as corrected for multiple comparisons using Monte Carlo simulations of the cluster size.

| **Max.** | **No. Vtx. Max.** | **Size (mm^2^)** | **MNI (X)** | **MNI (Y)** | **MNI (Z)** | **CWP** | **CWP (Low)** | **CWP (High)** | **No. Vtxs.** | **Weight Vtx.** | **Brain regions** |
| --- | --- | --- | --- | --- | --- | --- | --- | --- | --- | --- | --- |
| **Left cortical volume** | | | | | | | | | | | |
| -4.166 | 102,027 | 1,615 | -6 | -73 | 0 | 0.0001* | 0 | 0.0002 | 2,778 | -5,479 | Cuneus, calcarine sulcus, LingG, ventral PCC |
| **Right cortical volume** | | | | | | | | | | | |
| -3.958 | 62,792 | 2,687 | 16 | -94 | -10 | 0.0001* | 0 | 0.0002 | 3,820 | -7,173 | Cuneus, calcarine sulcus, occipital pole, POS |
| -3.334 | 68,910 | 823 | 16 | -88 | 19 | 0.0281 | 0.026 | 0.0302 | 1,082 | -2,117 | Occipital pole, SOG |
| **Left cortical surface area** | | | | | | | | | | | |
| -2.830 | 69,523 | 1,491 | -8 | -50 | 13 | 0.0009* | 0.0005 | 0.0013 | 3,130 | -5,826 | LingG, calcarine sulcus, ventral PCC |
| 4.510 | 18,380 | 1,292 | -60 | -51 | 17 | 0.0025* | 0.0019 | 0.0031 | 2,844 | 6,627 | STS, planum temporale, SupraMG, AngG |
| **Right cortical surface area** | | | | | | | | | | | |
| -3.145 | 117,795 | 1,563 | 14 | -91 | 19 | 0.0004* | 0.0002 | 0.0007 | 2,127 | -4,139 | Occipital pole, POS, SOG, cuneus |
| **Left cortical thickness** | | | | | | | | | | | |
| -3.224 | 9,559 | 847 | -30 | -68 | 25 | 0.0110* | 0.0097 | 0.0123 | 1,468 | -2,767 | Occipital pole, MOG, SOS, IPS |
| 3.635 | 6,655 | 711 | -10 | 53 | -22 | 0.0352 | 0.0328 | 0.0376 | 1,143 | 2,299 | OFC, rectal gyrus |
| **Right cortical thickness** | | | | | | | | | | | |
| 3.406 | 79,063 | 718 | 42 | -3 | -20 | 0.0333 | 0.031 | 0.0356 | 1,657 | 3,454 | Temporal pole, planum polare, AIC |
| **Left local gyrification index** | | | | | | | | | | | |
| 2.992 | 73,390 | 12,701 | -51 | 21 | 18 | 0.0001* | 0 | 0.0002 | 29,445 | 54,799 | STG, STS, HG, insula, SubCG, PreCG, CS, PostCG, PostCS, AngG, SupraMG opercular/triangular IFG, IFS, MFG |
| **Right local gyrification index** | | | | | | | | | | | |
| 3.869 | 75,834 | 6,272 | 51 | -24 | -13 | 0.0001* | 0 | 0.0002 | 13,913 | 24,769 | MTG, STS, STG, HG, insula, SubCG, PostCG, opercular/triangular IFG, IFS |
| 2.963 | 242 | 3,587 | 40 | 20 | 46 | 0.0001* | 0 | 0.0002 | 7,387 | 12,967 | MFG, IFS, PreCG, CS, PostCG, PostCS |
| 2.169 | 24,994 | 841 | 18 | 46 | -15 | 0.0454 | 0.0427 | 0.0481 | 1,442 | 2,199 | OFC, rectal gyrus |

Abbreviations: *, p-value surviving Bonferroni correction for the four different measures (p<0.0125). AIC, anterior insular cortex; AngG, angular gyrus; CWP, cluster-wise p-value; CWP Low and CWP High, 90% confidence interval for CWP; CS, central sulcus; HG, Heschl’s gyrus; IFG, inferior frontal gyrus; IFS, inferior frontal sulcus; IPS, intraparietal sulcus; LingG, lingual gyrus; Max., maximum –log10(p*-*value) in the cluster (positive/negative values represent positive/negative correlations); MFG, middle frontal gyrus; MNI (XYZ), the Montreal Neurological Institute coordinates of the maximum; MOG, middle occipital gyrus; MTG, middle temporal gyrus; No. Vtx. Max., vertex number at the maximum; No. Vtxs., number of cluster vertices; OFC, orbitofrontal cortex; PCC, posterior cingulate cortex; POS, parieto-occipital sulcus; PostCG, postcentral gyrus; PostCS, postcentral sulcus; PreCG, precentral gyrus; Size, cluster size in mm^2^; SOG, superior occipital gyrus; SOS, superior occipital sulcus; STG, superior temporal gyrus; STS, superior temporal sulcus; SubCG, subcentral gyrus; SupraMG, supramarginal gyrus; Vtx., vertex; Vtxs., vertices; Weight Vtx., weight of cluster (size x intensity).

**3.5. Supplementary Table S5. Clusters of different cortical morphological measures associated with the linguistic control formal thought disorder dimension.** Clusters represent partial correlations corrected for sex, age, negative formal thought disorder dimension, scanner, medication, and corresponding global measures as well as corrected for multiple comparisons using Monte Carlo simulations of the cluster size.

| **Max.** | **No. Vtx. Max.** | **Size (mm^2^)** | **MNI (X)** | **MNI (Y)** | **MNI (Z)** | **CWP** | **CWP (Low)** | **CWP (High)** | **No. Vtxs.** | **Weight Vtx.** | **Brain regions** |
| --- | --- | --- | --- | --- | --- | --- | --- | --- | --- | --- | --- |
| **Left cortical volume** | | | | | | | | | | | |
| -3.069 | 137,313 | 1,207 | -7 | -71 | -1 | 0.0021* | 0.0015 | 0.0027 | 1,986 | -3,424 | Cuneus, calcarine sulcus, LingG |
| 3.763 | 58,701 | 1,012 | -59 | -51 | 20 | 0.0067* | 0.0057 | 0.0078 | 2,198 | 4,340 | STS, SupraMG, AngG |
| **Right cortical volume** | | | | | | | | | | | |
| -2.933 | 125,896 | 1,521 | 14 | -79 | 12 | 0.0001* | 0 | 0.0002 | 2,051 | -3,648 | Cuneus, calcarine sulcus |
| **Left cortical surface area** | | | | | | | | | | | |
| 4.021 | 147,033 | 1,423 | -58 | -53 | 18 | 0.0013* | 0.0009 | 0.0018 | 3,088 | 6,734 | STS, SupraMG, AngG |
| -3.111 | 95,479 | 1,194 | -20 | -49 | -4 | 0.0047* | 0.0038 | 0.0056 | 2,536 | -4,785 | LingG, ventral PCC, POS |
| Right cortical surface area – No significant clusters | | | | | | | | | | | |
| **Left cortical thickness** | | | | | | | | | | | |
| 5.492 | 67,827 | 743 | -9 | 53 | -22 | 0.0273 | 0.0252 | 0.0294 | 1,121 | 2,683 | OFC, rectal gyrus |
| -2.735 | 128,116 | 692 | -11 | -77 | 4 | 0.0423 | 0.0397 | 0.0449 | 1,019 | -1,920 | Cuneus, calcarine sulcus, LingG |
| 3.520 | 131,504 | 676 | -13 | 63 | 10 | 0.0477 | 0.045 | 0.0504 | 1,009 | 2,102 | Medial SFG |
| **Right cortical thickness** | | | | | | | | | | | |
| 3.422 | 42,710 | 971 | 46 | -5 | -18 | 0.0037* | 0.0029 | 0.0045 | 2,282 | 4,773 | Planum polare, anterior STG, AIC |
| **Left local gyrification index** | | | | | | | | | | | |
| 3.614 | 150,611 | 4,243 | -63 | -10 | 25 | 0.0001* | 0 | 0.0002 | 9,863 | 17,154 | SubCG, PreCG, CS, PostCG, SupraMG, planum temporale |
| 2.856 | 65,645 | 1,011 | -41 | 35 | 25 | 0.0128 | 0.0114 | 0.0143 | 1,632 | 2,839 | Opercular IFG, IFS, MFG |
| **Right local gyrification index** | | | | | | | | | | | |
| 3.692 | 34,911 | 6,100 | 44 | 20 | 19 | 0.0001* | 0 | 0.0002 | 12,834 | 23,397 | MTG, STS, STG, HG, insula, SubCG, PostCG, SupraMG, opercular/triangular IFG, IFS, MFG |
| 3.796 | 118,094 | 3,443 | 20 | 17 | -21 | 0.0001* | 0 | 0.0002 | 6,370 | 12,932 | AIC, orbital IFG, OFC, rectal gyrus, frontal pole, ventral ACC |
| 2.932 | 55,945 | 1,362 | 34 | -12 | -33 | 0.0013* | 0.0009 | 0.0018 | 2,464 | 4,049 | Fusiform gyrus, parahippocampal gyrus, LingG |

Abbreviations: *, p-value surviving Bonferroni correction for the four different measures (p<0.0125). ACC, anterior cingulate cortex; AIC, anterior insular cortex; AngG, angular gyrus; CS, cenral sulcus; CWP, cluster-wise p-value; CWP Low and CWP High, 90% confidence interval for CWP; HG, Heschl,s gyurs; IFG, inferior frontal gyrus; IFS, inferior frontal sulcus; LingG, lingual gyrus; Max., maximum –log10(p*-*value) in the cluster (positive/negative values represent positive/negative correlations); MFG, middle frontal gyrus; MNI (XYZ), the Montreal Neurological Institute coordinates of the maximum; MTG, middle temporal gyrus; No. Vtx. Max., vertex number at the maximum; No. Vtxs., number of cluster vertices; OFC, orbitofrontal cortex; PCC, posterior cingulate cortex; POS, parieto-occipital sulcus; PostCG, postcentral gyrus; PreCG, precentral gyrus; SFG, superior frontal gyrus; Size, cluster size in mm^2^; STG, superior temporal gyrus; STS, superior temporal sulcus; SubCG, subcentral gyrus; SupraMG, supramarginal gyrus; Vtx., vertex; Vtxs., vertices; Weight Vtx., weight of cluster (size x intensity).

**3.6. Supplementary Table S6. Clusters of different cortical morphological measures associated with the negative formal thought disorder dimension.** Clusters represent partial correlations corrected for sex, age, positive formal thought disorder dimension, scanner, medication, and corresponding global measures as well as corrected for multiple comparisons using Monte Carlo simulations of the cluster size.

| **Max.** | **No. Vtx. Max.** | **Size (mm^2^)** | **MNI (X)** | **MNI (Y)** | **MNI (Z)** | **CWP** | **CWP (Low)** | **CWP (High)** | **No. Vtxs.** | **Weight Vtx.** | **Brain regions** |
| --- | --- | --- | --- | --- | --- | --- | --- | --- | --- | --- | --- |
| Left cortical volume – No significant clusters | | | | | | | | | | | |
| **Right cortical volume** | | | | | | | | | | | |
| -3.448 | 129,181 | 979 | 24 | -98 | -5 | 0.0091* | 0.0079 | 0.0103 | 1,240 | -2,586 | Occipital pole |
| Left cortical surface area – No significant clusters | | | | | | | | | | | |
| **Right cortical surface area** | | | | | | | | | | | |
| -4.542 | 125,808 | 2,609 | 15 | -99 | 12 | 0.0001* | 0 | 0.0002 | 3,372 | -6,780 | Occipital pole, SOG, cuneus |
| Left cortical thickness – No significant clusters | | | | | | | | | | | |
| Right cortical thickness – No significant clusters | | | | | | | | | | | |
| **Left local gyrification index** | | | | | | | | | | | |
| -3.971 | 159,106 | 5,489 | -13 | -51 | 40 | 0.0001* | 0 | 0.0002 | 10,915 | -19,755 | LingG, calcarine sulcus, cuneus, POS, precuneus, dorsal PCC |
| -2.168 | 64,260 | 1,701 | -8 | 51 | -6 | 0.0001* | 0 | 0.0002 | 2,887 | -4,772 | OFC, rectal gyrus, ventral ACC |
| **Right local gyrification index** | | | | | | | | | | | |
| -3.073 | 104,309 | 4,378 | 13 | -63 | 27 | 0.0001* | 0 | 0.0002 | 6,602 | -11,294 | Occipital pole, LingG, calcarine sulcus, cuneus, POS, precuneus |
| -2.989 | 123,518 | 1,647 | 13 | 12 | 40 | 0.0001* | 0 | 0.0002 | 3,696 | -6,518 | Middle ACC, medial SFG |
| -2.651 | 98,944 | 1,237 | 41 | -69 | -15 | 0.0029* | 0.0022 | 0.0036 | 1,871 | -3,397 | Fusiform gyrus, IOG, ITS |
| -2.019 | 56,460 | 1,011 | 33 | -48 | 38 | 0.0148 | 0.0133 | 0.0164 | 2,494 | -3,837 | SPL, IPS |

Abbreviations: *, p-value surviving Bonferroni correction for the four different measures (p<0.0125). ACC, anterior cingulate cortex; CWP, cluster-wise p-value; CWP Low and CWP High, 90% confidence interval for CWP; IOG, inferior occipital sulcus; IPS, intraparietal sulcus; ITS, inferior temporal sulcus; LingG, lingual gyrus; Max., maximum –log10(p*-*value) in the cluster (positive/negative values represent positive/negative correlations); MNI (XYZ), the Montreal Neurological Institute coordinates of the maximum; No. Vtx. Max., vertex number at the maximum; No. Vtxs., number of cluster vertices; OFC, orbitofrontal cortex; PCC, posterior cingulate cortex; POS, parieto-occipital sulcus; Size, cluster size in mm^2^; SFG, superior frontal gyrus; SOG, superior occipital gyrus; SPL, superior parietal lobule; Vtx., vertex; Vtxs., vertices; Weight Vtx., weight of cluster (size x intensity).

**3.7. Supplementary Table S7. Clusters of different cortical morphological measures associated with general formal thought disorder.** Clusters represent partial correlations corrected for sex, age, scanner, medication, and corresponding global measures as well as corrected for multiple comparisons using Monte Carlo simulations of the cluster size.

| **Max.** | **No. Vtx. Max.** | **Size (mm^2^)** | **MNI (X)** | **MNI (Y)** | **MNI (Z)** | **CWP** | **CWP (Low)** | **CWP (High)** | **No. Vtxs.** | **Weight Vtx.** | **Brain regions** |
| --- | --- | --- | --- | --- | --- | --- | --- | --- | --- | --- | --- |
| **Left cortical volume** | | | | | | | | | | | |
| -4.216 | 102,026 | 1,688 | -6 | -72 | 1 | 0.0001* | 0 | 0.0002 | 2,918 | -5,925 | Cuneus, calcarine sulcus, LingG, ventral PCC |
| 4.206 | 1,914 | 1,078 | -60 | -51 | 19 | 0.0044* | 0.0036 | 0.0053 | 2,386 | 5,055 | STS, planum temporale, SupraMG, AngG |
| **Right cortical volume** | | | | | | | | | | | |
| -4.608 | 62,793 | 2,988 | 17 | -94 | -10 | 0.0001* | 0 | 0.0002 | 4,240 | -8,230 | Cuneus, calcarine sulcus, occipital pole, POS |
| -3.496 | 68,912 | 879 | 17 | -89 | 19 | 0.0197 | 0.0179 | 0.0215 | 1,154 | -2,332 | Occipital pole, SOG |
| **Left cortical surface area** | | | | | | | | | | | |
| -3.017 | 6,388 | 1,574 | -18 | -55 | -3 | 0.0004* | 0.0002 | 0.0007 | 3,296 | -6,431 | LingG, calcarine sulcus, ventral PCC |
| 5.062 | 106,811 | 1,473 | -59 | -51 | 17 | 0.0010* | 0.0006 | 0.0014 | 3,181 | 7,813 | STS, planum temporale, SupraMG, AngG |
| **Right cortical surface area** | | | | | | | | | | | |
| -3.468 | 117,794 | 1,675 | 15 | -91 | 19 | 0.0001* | 0 | 0.0002 | 2,279 | -4,558 | Cuneus, occipital pole, SOG, POS |
| **Left cortical thickness** | | | | | | | | | | | |
| 4.170 | 67,833 | 732 | -10 | 52 | -22 | 0.0294 | 0.0272 | 0.0316 | 1,142 | 2,432 | OFC, rectal gyrus |
| -3.227 | 9,559 | 688 | -30 | -68 | 25 | 0.0439 | 0.0413 | 0.0465 | 1,234 | -2,310 | MOG, SOS, IPS |
| **Right cortical thickness** | | | | | | | | | | | |
| 3.213 | 55,847 | 707 | 40 | -3 | -19 | 0.0383 | 0.0359 | 0.0408 | 1,667 | 3,318 | Temporal pole, planum polare, insula |
| -4.498 | 61,384 | 693 | 8 | -84 | 9 | 0.0437 | 0.0411 | 0.0463 | 932 | -2,183 | Cuneus, calcarine sulcus |
| **Left local gyrification index** | | | | | | | | | | | |
| 2.905 | 44,970 | 10,833 | -52 | -15 | 16 | 0.0001* | 0 | 0.0002 | 25,163 | 43,969 | STS, STG, HG, insula, SubCG, PreCG, CS, PostCG, PostCS, SupraMG, AngG, opercular/triangular IFG, IFS, MFG |
| **Right local gyrification index** | | | | | | | | | | | |
| 3.900 | 119,695 | 9,203 | 45 | 16 | 20 | 0.0001* | 0 | 0.0002 | 19,883 | 34,873 | MTG, STS, STG, HG, insula, SubCG, SupraMG, PostCS, PostCG, CS, PreCG, opercular/triangular IFG, IFS, MFG |
| 2.412 | 24,994 | 1,199 | 18 | 46 | -15 | 0.0037* | 0.0029 | 0.0045 | 2,075 | 3,358 | OFC, rectal gyrus, ventral ACC |

Abbreviations: *, p-value surviving Bonferroni correction for the four different measures (p<0.0125). ACC, anterior cingulate cortex; AngG, angular gyrus; CS, central sulcus; CWP, cluster-wise p-value; CWP Low and CWP High, 90% confidence interval for CWP; Max., maximum –log10(p*-*value) in the cluster (positive/negative values represent positive/negative correlations); HG, Heschl’s gyrus; IFG, inferior frontal gyrus; IFS, inferior frontal sulcus; IPS, intraparietal sulcus; LingG, lingual gyrus; MFG, middle frontal gyrus; MNI (XYZ), the Montreal Neurological Institute coordinates of the maximum; MOG, middle occipital gyrus; MTG, middle temporal gyrus; No. Vtx. Max., vertex number at the maximum; No. Vtxs., number of cluster vertices; OFC, orbitofrontal cortex; PCC, posterior cingulate cortex; POS, parieto-occipital sulcus; PostCG, postcentral gyrus; PostCS, postcentral sulcus; PreCG, precentral gyrus; Size, cluster size in mm^2^; SOG, superior occipital gyrus; SOS, superior occipital sulcus; SubCG, subcentral gyrus; STG, superior temporal gyrus; STS, superior temporal sulcus; SupraMG, supramarginal gyrus; Vtx., vertex; Vtxs., vertices; Weight Vtx., weight of cluster (size x intensity).

**
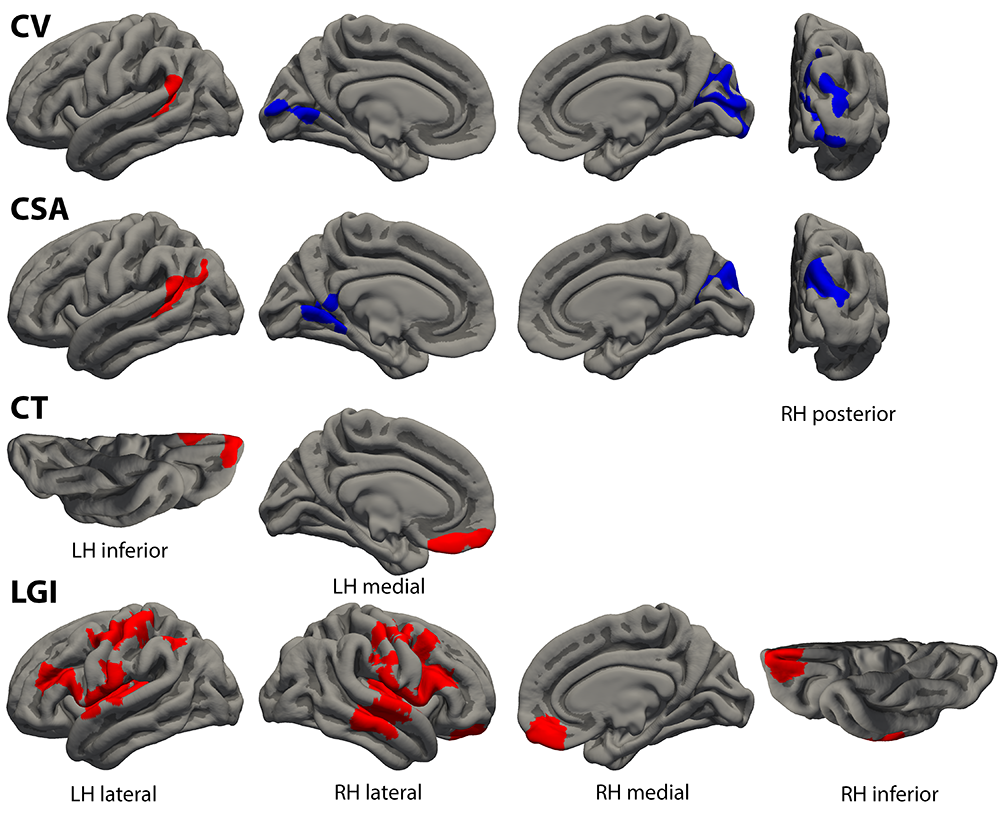
**

**3.8. Supplementary Figure S1. Clusters of different cortical morphological measures associated with the sum score of the thought, language, and communication scale.** Clusters represent partial correlations (positive in red, negative in blue) corrected for sex, age, scanner, and corresponding global measures as well as corrected for multiple comparisons using Monte Carlo simulations of the cluster size. Cluster information is summarized in Supplementary Table S3. Abbreviations: CSA, cortical surface area; CT, cortical thickness; CV, cortical volume; LGI, local gyrification index; LH, left hemisphere; RH, right hemisphere.

1. **Supplementary References**

1 Dale, A. M., Fischl, B. & Sereno, M. I. Cortical surface-based analysis. I. Segmentation and surface reconstruction. *NeuroImage* **9**, 179-194 (1999). <https://doi.org:10.1006/nimg.1998.0395>

2 Fischl, B., Sereno, M. I. & Dale, A. M. Cortical surface-based analysis. II: Inflation, flattening, and a surface-based coordinate system. *NeuroImage* **9**, 195-207 (1999). <https://doi.org:10.1006/nimg.1998.0396>

3 Fischl, B., Liu, A. & Dale, A. M. Automated manifold surgery: constructing geometrically accurate and topologically correct models of the human cerebral cortex. *IEEE Trans Med Imaging* **20**, 70-80 (2001). <https://doi.org:10.1109/42.906426>

4 Fischl, B. & Dale, A. M. Measuring the thickness of the human cerebral cortex from magnetic resonance images. *PNAS* **97**, 11050-11055 (2000). <https://doi.org:10.1073/pnas.200033797>

5 Rosas, H. D. *et al.* Regional progressive thinning of cortical ribbon in Huntington's disease. *Neurology* **58**, 695-701 (2002).

6 Kuperberg, G. R. *et al.* Regionally localized thinning of the cerebral cortex in schizophrenia. *Arch Gen Psychiatry* **60**, 878-888 (2003). <https://doi.org:10.1001/archpsyc.60.9.878>

7 Salat, D. H. *et al.* Thinning of the cerebral cortex in aging. *Cereb Cortex* **14**, 721-730 (2004). <https://doi.org:10.1093/cercor/bhh032>
